# Sea Spray Aerosol (SSA) as a Source of Perfluoroalkyl Acids (PFAAs) to the Atmosphere: Field Evidence from Long-Term Air Monitoring

**DOI:** 10.1021/acs.est.1c04277

**Published:** 2021-12-15

**Authors:** Bo Sha, Jana H. Johansson, Peter Tunved, Pernilla Bohlin-Nizzetto, Ian T. Cousins, Matthew E. Salter

**Affiliations:** † Department of Environmental Science, Stockholm University, SE-106 91 Stockholm, Sweden; ‡ Bolin Centre for Climate Research, SE-106 91 Stockholm, Sweden; § NILU - Norwegian Institute for Air Research, P.O. Box 100, 2027 Kjeller, Norway

**Keywords:** per- and polyfluoroalkyl substances (PFAS), perfluoroalkyl acids (PFAAs), sea spray aerosols (SSA), coastal areas, long-range atmospheric transport, air monitoring, Arctic, Norway

## Abstract

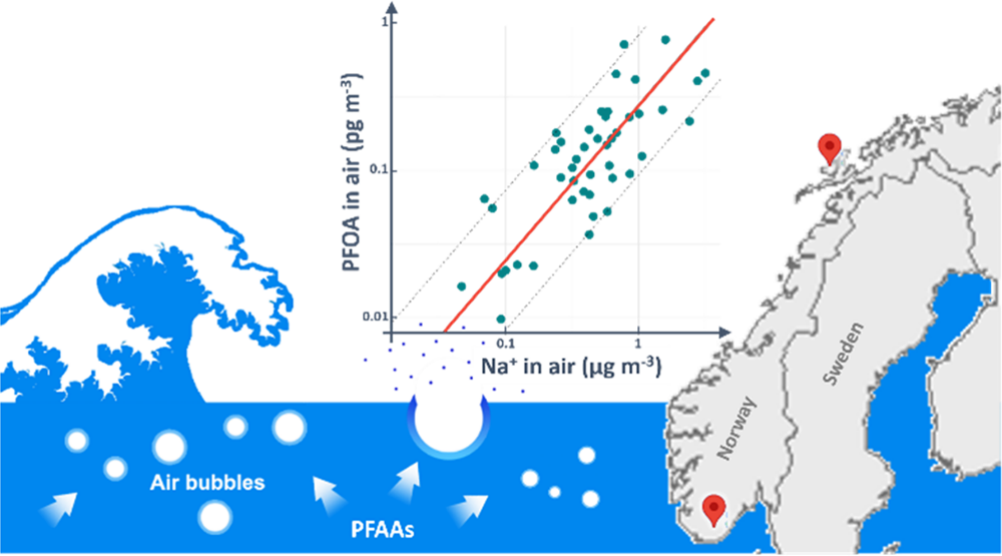

The effective enrichment of perfluoroalkyl acids (PFAAs) in sea spray aerosols (SSA) demonstrated in previous laboratory studies suggests that SSA is a potential source of PFAAs to the atmosphere. In order to investigate the influence of SSA on atmospheric PFAAs in the field, 48 h aerosol samples were collected regularly between 2018 and 2020 at two Norwegian coastal locations, Andøya and Birkenes. Significant correlations (*p* < 0.05) between the SSA tracer ion, Na^+^, and PFAA concentrations were observed in the samples from both locations, with Pearson’s correlation coefficients (*r*) between 0.4–0.8. Such significant correlations indicate SSA to be an important source of atmospheric PFAAs to coastal areas. The correlations in the samples from Andøya were observed for more PFAA species and were generally stronger than in the samples from Birkenes, which is located further away from the coast and closer to urban areas than Andøya. Factors such as the origin of the SSA, the distance of the sampling site to open water, and the presence of other PFAA sources (e.g., volatile precursor compounds) can have influence on the contribution of SSA to PFAA in air at the sampling sites and therefore affect the observed correlations between PFAAs and Na^+^.

## Abstract 2

The field evidence presented in the present study shows sea spray aerosols (SSA) can be an important source of atmospheric perfluoroalkyl acids (PFAAs) in coastal areas.

## 1 Introduction

Perfluoroalkyl acids (PFAAs), including perfluoroalkyl carboxylic acids (PFCAs) and perfluoroalkanesulfonic acids (PFSAs), are a group of persistent organic contaminants that have been found worldwide in abiotic environments, biota, and humans.^1−9^ Long-range atmospheric transport is considered to substantially contribute to the ubiquitous presence of PFAAs,^3,10^ especially in remote areas such as the Arctic and Antarctic. There are three major sources of PFAAs to the atmosphere: (1) direct emission from manufacturing sources such as fluoropolymer plants,^11,12^ (2) formation in the atmosphere via degradation from volatile precursors like fluorotelomer alcohols (FTOHs),^13,14^ and (3) water-to-air transfer via sea spray aerosol (SSA) emission.^15,16^

SSA is emitted at the sea surface by bubble bursting.^17^ When air is entrained into seawater by breaking waves, surface active substances such as PFAAs can be scavenged by the air–water interface of the bubbles and transferred to the atmosphere via SSA emission.^17^ Laboratory SSA simulation experiments have demonstrated that PFAA concentrations in submicron SSA can be 4–5 orders of magnitude higher than in the bulk water.^15,18^ On the basis of laboratory-derived enrichment factors (EFs) and reported median concentrations in seawater, the estimated fluxes of perfluorooctanoic acid (PFOA) and perfluorooctanesulfonic acid (PFOS) from SSA to the atmosphere were comparable with the other two sources of atmospheric PFAAs (i.e., direct emission from manufacturing sources and degradation from volatile precursors),^15^ suggesting the potential of SSA as an important source of PFAAs to the atmosphere.

PFAAs can travel a great distance in the atmosphere via SSA. For SSA particles with *r*_80_ (radius at 80% relative humidity) = 1, 2, and 5 μm, the estimated residence times with respect to dry deposition are ∼1.5 weeks, 2.3 days, and 10 h, and the transport distances are ∼10^4^, 2000, and 330 km, respectively.^19^ The removal of SSA by wet deposition depends on the frequency of precipitation events, which is usually several days to a week in a marine environment.^19^ The modeling results by Johansson et al. showed that the deposition of PFOA and PFOS to terrestrial environments via SSA transport may impact large areas of inland Europe and not only coastal areas.^15^

Despite increasing evidence that SSA may be an important source to the atmosphere from these laboratory studies,^15,16,18,20^ evidence from the field remains elusive. Long-term air monitoring between 2006 and 2014 showed significantly higher PFOA concentrations at two Norwegian Arctic stations, Zeppelin and Andøya, compared to the Canadian High Arctic station of Alert.^21^ It was speculated that the two Norwegian stations might receive additional PFOA transported via SSA since they are closer to open water (Andøya ∼1.3 km and Zeppelin ∼ 2km) than Alert (∼4 km from the coast and the surrounding water was covered by sea ice for most of the year).^21^ However, the PFOS concentrations were not significantly different between the three Arctic stations.^21^ It is hypothesized that, if SSA was an important source of PFAAs to the atmosphere, significant correlations between the concentrations of SSA tracer ions (e.g., Na^+^) and PFAAs should be observed in aerosol samples obtained in the marine atmosphere or at coastal locations where the atmospheric burden of SSA is highest. Casas et al. collected seven aerosol samples at a site located at South Bay, Livingston Island, Antarctica, but observed no such correlation in the samples,^9^ which might be due to the small sample size collected within a relatively short time period (one month).

Atmospheric deposition samples such as surface snow have also been used to examine the relationship between PFAAs and SSA. Kwok et al. collected surface snow samples (*n* = 10) around the coastal area of Longyearbyen, Svalbard Archipelago, Norway, in May 2006 and found that the concentrations of the sea-salt component SO_4_^2–^ were significantly correlated with perfluorohexanesulfonic acid (PFHxS) and PFOS concentrations (*r* > 0.9, *p* < 0.0001) but not with PFCA concentrations.^22^ A series of field studies have investigated snow pit samples or ice core samples from ice caps that represented atmospheric deposition from multiple years and found no correlations between the concentrations of PFAAs and SSA tracer ions.^22−25^ The lack of correlation may be because SSA only contributed to a small portion of the PFAA deposition on the high altitude ice caps. Ice core samples represent atmospheric deposition from multiple years or decades. Compared with annual PFAA and PFAA-precursor emissions,^26,27^ the annual SSA deposition is expected to be less variable over decades. So the changes in yearly PFAA deposition may be mainly driven by the changes in historical PFAA emissions rather than SSA deposition. In addition, other factors such as melting events during a temporary warm period can lead to uncertainty^25^ when using deposition samples to examine the links between PFAAs and SSA tracer ions. Therefore, direct field evidence is still needed to understand the influence of SSA on PFAAs in the atmosphere.

In this study, air sampling was conducted regularly at two Norwegian coastal sites across a two-year period in order to obtain aerosol samples with a wide range of SSA loading. The aim was to establish a long-term and large data set in which PFAAs and SSA tracer ions were measured in the same samples so that the correlations between the concentrations of PFAAs and SSA tracer ions could be determined to help improve our understanding of the importance of SSA as a source of PFAAs to the atmosphere.

## 2 Material and Methods

### 2.1 Target Compounds

A total of 11 PFAAs were investigated in this study, including C5–C12 PFCAs and C4, C6, and C8 PFSAs (“C*n*” indicates the total number of carbon atoms). The PFCAs were perfluoropentanoic acid (PFPeA), perfluorohexanoic acid (PFHxA), perfluoroheptanoic acid (PFHpA), PFOA, perfluorononanoic acid (PFNA), perfluorodecanoic acid (PFDA), perfluoroundecanoic acid (PFUnDA), and perfluorododecanoic acid (PFDoDA) and the PFSAs were perfluorobutanesulfonic acid (PFBS), PFHxS, and PFOS. Details of the target compounds, analytical standards, and reagents used can be found in Tables S1 and S2 in the Supporting Information. Sodium (Na^+^) and magnesium (Mg^2+^) ions were analyzed as tracers of SSA.

### 2.2 Aerosol Sampling

Ambient aerosol samples were collected at two Norwegian coastal sites, Andøya (69°16′N, 16°00′E, 380 m above sea level, and ∼1.3 km to open water) and Birkenes (58°23′N, 8°15′E, 190 m above sea level, and ∼20 km to open water), as shown in Figure S1. The sampling at Andøya (*n* = 57) was carried out between April 2018 and July 2020 with 2–3 samples taken per month. The samples from Birkenes (*n* = 58) were collected at a higher frequency of about 5–8 samples per month between April and October in 2018 and between September and December in 2019. The samples were collected on prebaked (800 °C for 8 h) quartz fiber filters (QFFs, φ = 150 mm, MK360, Munktell) using a Digitel (DH77) high-volume active air sampler (HV-AAS) operated at a flow rate of ∼500 L min^–1^. For each sample, ∼1500 m^3^ air was collected over 48 h, with a few exceptions. A complete sample list including the start date, duration, and sample volume is provided in Table S3.

### 2.3 Sample Preparation and Analysis

Prior to extraction, a small hole (φ = 13 mm) was punched in each QFF. The punch was stored for sodium (Na^+^) and magnesium (Mg^2+^) analysis. The rest of the filter was used for PFAA analysis. PFAAs were analyzed on an Acquity ultraperformance liquid chromatography system coupled to a Xevo TQ-S tandem mass spectrometer (UPLC/MS/MS; Waters Corp.) based on a previously published method.^28^ Na^+^ and Mg^2+^ ions were analyzed using an inductively coupled plasma-atomic emission spectrometer (ICP-AES, Spectro Cirros^CCD^, Kleve, Germany) located at the Department of Chemistry, Uppsala University, Sweden. Details regarding the extraction of the samples and the analysis of PFAAs can be found in the Supporting Information.

### 2.4 QA/QC

Field blanks were produced routinely at Andøya (*n* = 9) and Birkenes (*n* = 7) by loading the QFF on the HV-AAS and exposing them to ambient air for 1 min without turning the pump on. The field blanks were treated the same as the samples. Two blank QFFs (prebaked at 800 °C for 8 h) were extracted as laboratory blanks and analyzed with every 20 samples.

To check the distribution of SSA on the filters, triplicate punches were randomly taken on 10% of the samples (*n* = 12) and analyzed for sodium and magnesium. The relative standard deviations of triplicates were generally < 5%. Therefore, it can be assumed that SSA were evenly distributed on the filters and the small punches were representative of the whole filters. Details regarding the blank levels, MDLs, MQLs, IS recoveries, and spike-recovery test can be found in Section S2 and Table S4.

### 2.5 Air Mass Trajectory Analysis

The HYSPLIT4 model^29^ was used for air mass backward trajectory analysis, and the meteorological data set used was the one degree Global Data Assimilation System (GDAS1) archive (https://www.ready.noaa.gov/archives.php). The HYSPLIT model was run using the ensemble method 10 days (240 h) back in time. For each sample, the trajectory footprint was calculated and used to determine the transport probability function plot (P[A_*i,j*_]), which represents the probability of a backward trajectory passing through a certain grid cell (*i,j*). This was used to visualize the dominant movement path of the air masses for any given period. The footprint analysis of each sample was further used to estimate the source attribution function plot (*C̅*_*i*__,*j*_), which represents the average observed concentration of PFAAs and Na^+^ if the trajectory spent time in a specific grid cell (*i*,*j*). These source attribution function plots were used to identify potential source areas of PFAAs. Details regarding the trajectory analysis can be found in the Supporting Information.

### 2.6 Statistical Analysis

Statistical analysis was conducted using RStudio (Version 1.2.5033) with R version 3.6.3. Concentration data were logarithm-transformed (i.e., log_10_[PFAA] and log_10_[Na]) before statistical analysis was performed and values below the MDLs were not included, unless otherwise stated. Values between the MDLs and MQLs were included without adjustment. Log–log linear correlations were evaluated using the Pearson’s correlation coefficient (*r*). To prevent a few extreme values biasing the correlations, only samples with PFAA and Na^+^ concentrations > MDLs and PFAA/Na^+^ ratios between the 5th and 95th percentiles at each location were included (i.e., 90% of the samples). Correlation analysis was also performed including the data with <5th percentile and >95th percentile PFAA/Na^+^ values and substituting values < MDL by 1/2MDL. These different data treatments did not affect the significance of the correlations for PFSAs in the samples from both sites and C7–C10 PFCAs in the Andøya samples but did affect the PFCAs in the Birkenes samples. For example, when all samples were considered, none of the PFCAs (*p* > 0.05) were correlated with Na^+^ at Birkenes. Details about the results of the correlation analysis and orthogonal regression with different data treatments are included in Table S5.

## 3 Results and Discussion

### 3.1 PFAA Concentrations at Andøya and Birkenes

**Figure 1:**
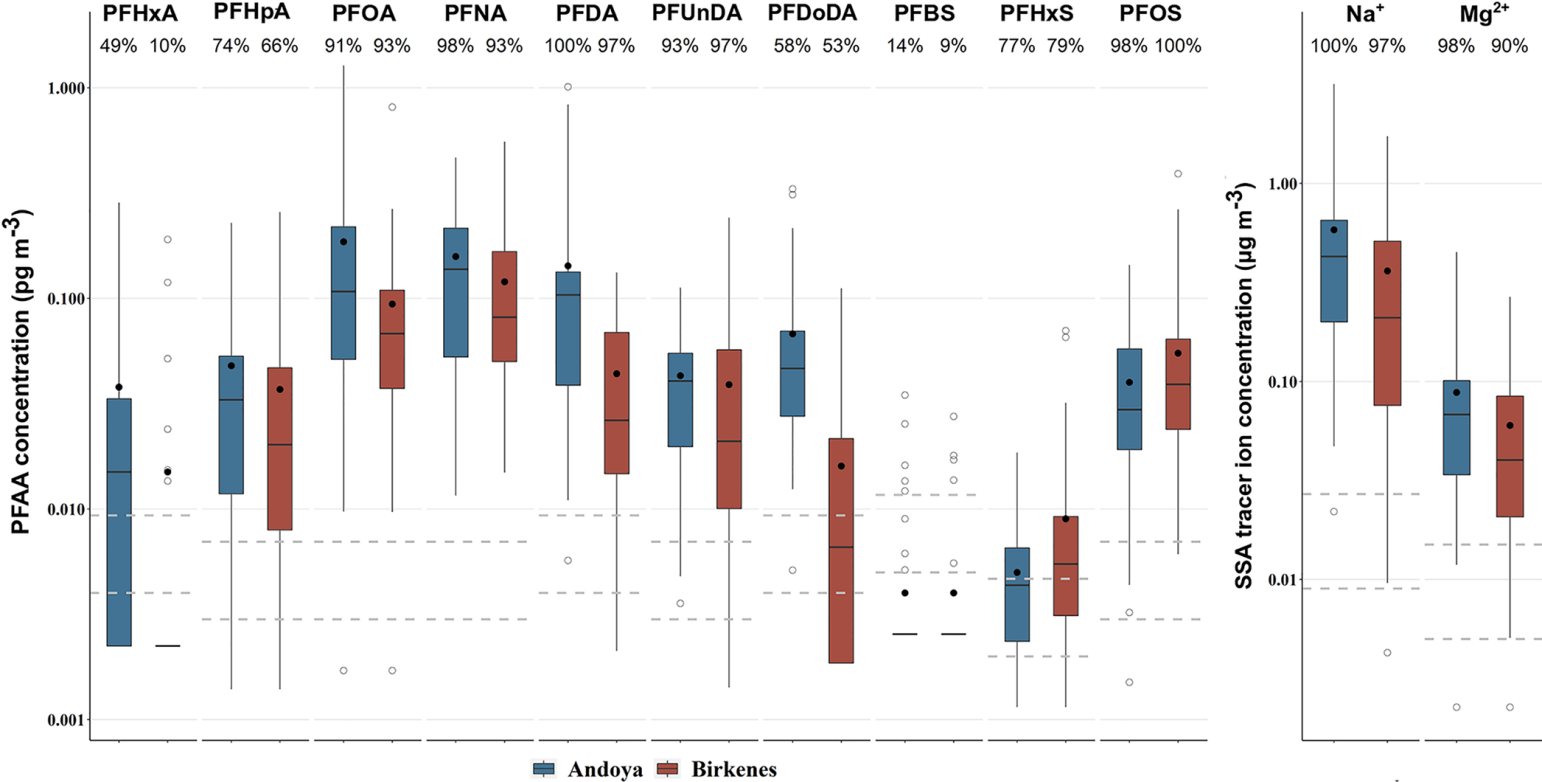
Box-whisker plot of concentrations of PFAAs, Na^+^, and Mg^2+^ in the air samples from Andøya and Birkenes. The lower and upper hinges correspond to the first and third quartiles (the 25th and 75th percentiles). The black lines and dots inside the boxes indicate the medians and means, respectively. Values below the MDLs were replaced by 1/2MDLs. The whiskers extend to no further than 1.5*IQR (interquartile range) from the hinges. The circles indicate values outside of 1.5*IQR. The gray dashed lines indicate MDLs (lower) and MQLs (upper). The numbers below the compound names are detection frequencies at each of the two sampling locations.

PFAAs were detected in all samples, and the results are shown in Figure 1. Details regarding the detection frequencies (DF) of the target compounds, medians, ranges, etc. can be found in Table S6. C7–C12 PFCAs were frequently detected in samples from both Andøya (*n* = 57) and Birkenes (*n* = 58), with detection frequencies > 50%. PFHxA was found in 43% of the Andøya samples but in only 10% of the Birkenes samples. PFPeA was not reported due to a matrix effect which resulted in a high chromatographic baseline. The median concentrations of ∑PFCAs (where the total is the sum of C6–C12 PFCAs and values < MDL were replaced by 1/2MDL) were (ranges given in parentheses) 0.46 pg m^–3^ (0.045–3.4 pg m^–3^) at Andøya and 0.22 pg m^–3^ (0.012–2.0 pg m^–3^) at Birkenes. The concentrations of PFNA, PFDA, and ∑PFCAs were significantly higher in the Andøya samples than in the Birkenes samples (*t* test, *p* < 0.001).

For PFSAs, PFOS was detected in almost all samples and PFHxS was detected in >70% of the samples from both locations. PFBS was excluded from the following analysis due to its low detection frequency (<15% at both locations). The concentrations of ∑PFSAs (where total is the sum of PFHxS and PFOS and values < MDL were replaced by 1/2MDL) were about 1 order of magnitude lower than for ∑PFCAs, with median concentrations (ranges in parentheses) of 0.033 pg m^–3^ (0.004–0.16 pg m^–3^) and 0.045 pg m^–3^ (0.007–0.46 pg m^–3^) at Andøya and Birkenes, respectively. No significant differences (*p* > 0.05) were observed for PFSAs between the two sampling sites.

The concentrations of PFOA (median: 0.11 pg m^–3^ and range: < 0.003–1.3 pg m^–3^) and PFOS (median: 0.030 pg m^–3^ and range: < 0.003–0.14 pg m^–3^) at Andøya were comparable to previously reported data for samples collected between 2010–2014 at this site (median: 0.24 pg m^–3^ and range: < 0.12–5.5 pg m^–3^ for PFOA and median: 0.072 pg m^–3^ and range: < 0.043–0.43 pg m^–3^ for PFOS).^21^ The detection frequencies of PFCAs with more than 8 carbons (C > 8) at both locations in this study were much higher than reported in previous studies (<25%).^21,30,31^

Significant log–log linear correlations (*p* < 0.05) were observed between all possible pairs of PFCAs at both Andøya (*r* = 0.68–0.94) and Birkenes (*r* = 0.47–0.94) as shown in Figure S2. Additionally, PFHxS and PFOS were also found to be positively and significantly correlated with PFCAs (*r* = 0.2–0.68, *p* < 0.05) with only a few exceptions. The degradation of FTOHs in the atmosphere would form a series of PFCA homologues, while both PFCAs and PFSAs can be transported via SSA since they are all frequently detected in seawater.^32^ Although perfluoroalkyl sulfonyl fluoride (PASF)-based precursor compounds such as *x*-perfluorooctanesulfonamides/sulfonamido ethanols (xFOSAs/Es) can form both PFSAs and PFCAs in the atmosphere,^14^*x*FOSAs/Es were not frequently detected in the long-term air monitoring program at the two sampling sites (DF < 20%) and their concentrations were orders of magnitude lower than FTOHs.^30,31^ Therefore, such a correlation pattern may suggest the combined effect of both transport via SSA and degradation from FTOHs.

### 3.2 Na^+^ and Mg^2+^ Ion Concentrations at the Two Locations

The Na^+^ ion was detectable in almost all air samples (100% at Andøya and 97% at Birkenes). The Na^+^ ion concentrations in the samples from Andøya (median: 0.21 μg m^–3^ and range: 0.022–3.2 μg m^–3^) were significantly higher (*p* < 0.01) than in the samples from Birkenes (median: 0.04 μg m^–3^ and range: < 0.009–1.7 μg m^–3^, Figure 1 and Table S6). Significant correlations between Na^+^ and Mg^2+^ were observed at both locations (not logarithm-transformed, *r* = 0.99 at Andøya, and *r* = 0.98 at Birkenes, *p* < 0.001). The Mg^2+^/Na^+^ ratios determined by the slopes of the orthogonal regressions (Figure S3) were 0.135 and 0.141 at Andøya and Birkenes, respectively, which are close to the observed ratio of 0.119 in seawater.^33^ The good correlation between the two elements and the close Mg^2+^/Na^+^ ratio to the Mg^2+^/Na^+^ ratio of seawater suggest that sea spray aerosol was a major source of particulate matter at the two sampling sites.

### 3.3 Correlations between PFAAs and Na^+^ Air Concentrations at the Two Sampling Sites

**Figure 2:**
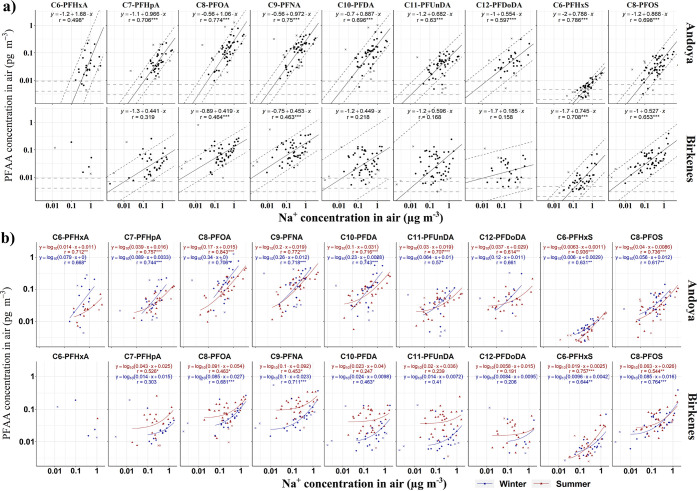
Correlations between PFAAs and Na^+^ in (a) air samples with PFAA/Na^+^ ratios between the 5th and 95th percentiles and (b) in summer and winter samples. The strength of the log–log linear correlation is indicated by the *r* value (Pearson’s correlation coefficient). The significance of the correlation is indicated by the number of asterisks (*p < 0.05, **p < 0.01, ***p < 0.001). Data not included in the correlation analysis are marked as “×”. In Figure 1a, the solid lines are fitted by orthogonal linear regression in the form of log_10_[PFAA] = *k* × log_10_[Na^+^] + *b* and the dashed lines in parallel represent ± σ. The gray horizontal dashed lines in panel (a) indicate MDLs (lower) and MQLs (upper) of individual PFAAs. In Figure 1b, the solid lines are fitted in the form of log_10_[PFAA] = log_10_(*k*_*SSA*_ × [Na^+^] + [PFAA]_other_).

Positive log–log linear correlations between PFAA and Na^+^ ion concentrations were observed in air samples from both locations (Figure 2a), suggesting that SSA can be an important source of PFAAs to the atmosphere in coastal areas. For the Andøya samples, all PFCAs (C6–C12) and PFSAs (C6 and C8) were found to be positively correlated with Na^+^ (*r* = 0.50–0.79, *p* < 0.05). PFOA showed the strongest correlations with Na^+^ (*r* = 0.77, *p* < 0.001) among the PFCAs. The correlations became weaker both as the chain length decreased and increased relative to PFOA. PFHxS (*r* = 0.79, *p* < 0.001) showed a better correlation with Na^+^ than PFOS (*r* = 0.70, *p* < 0.001). At Birkenes, PFOA (*r* = 0.46, *p* < 0.001) and PFNA (*r* = 0.46, *p* < 0.001) were the only PFCAs that correlated with Na^+^ and the correlations were weaker than at Andøya. In the case of the two PFSAs, the correlations with Na^+^ at Birkenes (*r* = 0.71 and 0.65 for PFHxS and PFOS, respectively, *p* < 0.001) were similar to those observed at Andøya.

The amount of PFAAs transported from the ocean to the atmosphere is determined by both their concentrations in seawater and the enrichment factor in SSA.^15,18^ Compared to PFOA and PFNA, laboratory studies have shown that C < 8 PFCAs have ∼3–20 fold lower enrichment factors^15,18^ while field measurements have shown that C > 9 PFCAs are usually present in seawater at concentrations orders of magnitude lower.^2,34−37^ Such laboratory and field observations may explain why PFOA and PFNA exhibit the strongest correlation with Na^+^ among the PFCAs at Andøya (*r* = 0.77 and 0.75 for PFOA and PFNA, respectively, *p* < 0.001) and why they were the only two PFCAs that showed significant correlations with Na^+^ at Birkenes (*r* = 0.46 for both, *p* < 0.001).

Different origins of SSA could be another factor influencing the correlation between PFAA and Na^+^. PFAA seawater concentrations vary over several orders of magnitude from populated coastal areas to the open ocean.^2,34−39^ SSA produced from areas with different PFAA concentrations would also vary in the amount of PFAA transferred to air per pg of SSA (Na^+^), which could weaken the correlations.

The potential influence of sources other than SSA (e.g., transformation of volatile precursors^14,21^) can also weaken the correlations observed between PFAAs and Na^+^. For example, if the fluctuation in air concentrations of precursor compounds and/or the yield of the transformation is large in relation to the contribution from SSA, the correlation between PFAAs and Na^+^ might be weak or even difficult to observe. Birkenes has a lower SSA burden than Andøya (lower Na^+^ concentration) and is closer to an urban area where consumer products containing PFCA precursor compounds were likely used widely.^40^ This may explain the weaker correlation with Na^+^ for PFOA and PFNA in the Birkenes samples (*r* < 0.5) and the lack of correlation with Na^+^ for the other PFCAs. Additionally, different SSA origin and influence of other sources can lead to variability in the [PFAA]_air_/[Na^+^]_air_ ratio and result in the slope of the log–log linear relationship deviating from 1. For example, for PFOA, the slope estimated for the Birkenes samples is 0.419 while the slope for the Andøya samples is 1.06 (Figure 2a).

In contrast to the PFCAs, the *r*-values of the correlations between Na^+^ and the two PFSAs did not differ much between Andøya (*r* = 0.79 and 0.70 for PFHxS and PFOS, respectively) and Birkenes (*r* = 0.71 and 0.65 for PFHxS and PFOS, respectively, Figure 2a). This suggests that for PFSAs, non-SSA sources are less important or make similar contributions at the two sites. Perfluorohexane sulfonyl fluoride (PHxSF)-based, precursors, which transform to PFHxS, were not intentionally produced in large quantities,^41^ and as mentioned previously, reported concentrations of major PFOS precursors, i.e., *x*FOSAs/Es, were not frequently detected (DF < 20%) at the two sampling sites.^30,31^ Thus, the observed correlations for PFSAs and low PFSA precursor concentrations in air suggest that the contribution from SSA may be more important for PFSAs compared to PFCAs.

Interestingly, while the overall PFNA and PFDA loadings were significantly higher in the Andøya samples (*t* test, *p* < 0.001, section 3.1), the ratios of PFNA/Na^+^, PFDA/Na^+^, and PFOS/Na^+^ were found to be significantly higher in the Birkenes samples (*p* < 0.05). SSA originating from more polluted areas (e.g., populated coastal areas) would have higher PFAA/Na^+^ ratios since laboratory results show that the amount of PFAAs transferred via SSA emission is positively correlated with their seawater concentrations.^18^ Additionally, greater contributions from other sources such as transformation from precursors may also lead to a higher PFAA/Na^+^ ratio.

### 3.4 Seasonal Differences

SSA production can be affected by a number of environmental variables including wind speed and the sea surface temperature.^17^ The formation of PFAAs via degradation from volatile precursors can also be affected by environmental variables including temperature, ultraviolet (UV) light intensity, and hydroxyl (OH) radical concentrations.^13,14^ These parameters all have a seasonal pattern, so the contribution of these two sources to atmospheric PFAAs may differ between seasons. To investigate whether the correlations between PFAAs and Na^+^ revealed seasonal differences, the samples from the two sampling sites were grouped as summer samples (collected between April first and September 30th) and winter samples (collected between October first and March 31st). Since the PFAA concentrations in air can be simplified as the combination of the contribution from SSA ([PFAA]_SSA_) and the contribution from other sources ([PFAA]_other_), in addition to log–log linear Pearson correlation analysis between PFAA and Na^+^, the data of the two seasons were fitted to a linear function of the form:
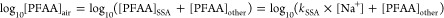
1where *k*_SSA_ (pg μg^–1^ Na^+^) represents the amount of PFAA transferred to the atmosphere per μg of Na^+^ and [PFAA]_other_ (pg m^–3^) represents the average of other sources. Results of the correlation analysis and the parameters of the fitted lines are included in Figure 2b and Table S7.

Almost all PFAAs were correlated with Na^+^ (*p* < 0.05) in both summer and winter samples collected at Andøya except PFDoDA, for which the log–log linear correlation was only observed in summer samples. For Andøya winter samples, the fitted lines for each PFCA have greater *k*_SSA_ (0.064–0.345) and lower [PFAA]_other_ (0–0.012) than those of the summer samples (*k*_SSA_ = 0.030–0.198 and [PFAA]_other_ = 0.011–0.031). In other words, the PFCA concentrations in air at Andøya during winter appear more sensitive to changes in Na^+^ concentrations, but the contribution from other sources was lower compared to summer. PFOS showed a similar pattern to PFCAs, but the difference between winter (*k*_SSA_ = 0.056, [PFAA]_other_ = 0.009, and *r* = 0.617) and summer (*k*_SSA_ = 0.040, [PFAA]_other_ = 0.012, and *r* = 0.736) was not as large as observed for PFCAs.

The lower contribution from other sources in winter may be the result of reduced yield of the indirect photolysis of PFAA precursors. In winter, the fewer daylight hours would lead to lower concentrations of OH radicals in the atmosphere, which would limit the reaction rate.^14,42,43^ Concentrations of PFAA precursors (e.g., FTOHs) in air were also found to be positively correlated with air temperature,^21^ and the lower air concentration of precursor compounds in winter would further limit the degradation process to form PFAAs.

For the Birkenes samples, correlations with Na^+^ were observed for C8–C10 PFCAs (*p* < 0.05) in the winter samples and for C7–C9 PFCAs (*p* < 0.05) in the summer samples. The two PFSAs were correlated with Na^+^ (*p* < 0.01) in both sample groups. Similar to the Andøya samples, [PFAA]_other_ obtained from the fitted lines for PFOA, PFNA, and PFOS at Birkenes were lower in winter than in summer, suggesting a lower contribution from other sources in winter than in summer. However, contrary to the Andøya samples, *k*_SSA_ of the Birkenes samples were similar between the seasons, implying little variation of the contribution from SSA. The longer distance to the coast (∼20 km) than Andøya (∼1.3 km) may have limited the size of SSA that can be transported to Birkenes, which may be one potential cause for the observed differences. However, since the PFAA enrichment in SSA under real-world conditions is still not very well-understood, it is difficult to conclude the causes of the observed seasonal differences. Further laboratory studies on the enrichment mechanisms of PFAAs in SSA (e.g., the influence of natural organic matter) and field studies with size-resolved aerosol sampling techniques will help improve the understanding of the contribution of SSA to the atmospheric transport of PFAAs.

### 3.5 Backward Air Mass Trajectory Analysis

**Figure 3:**
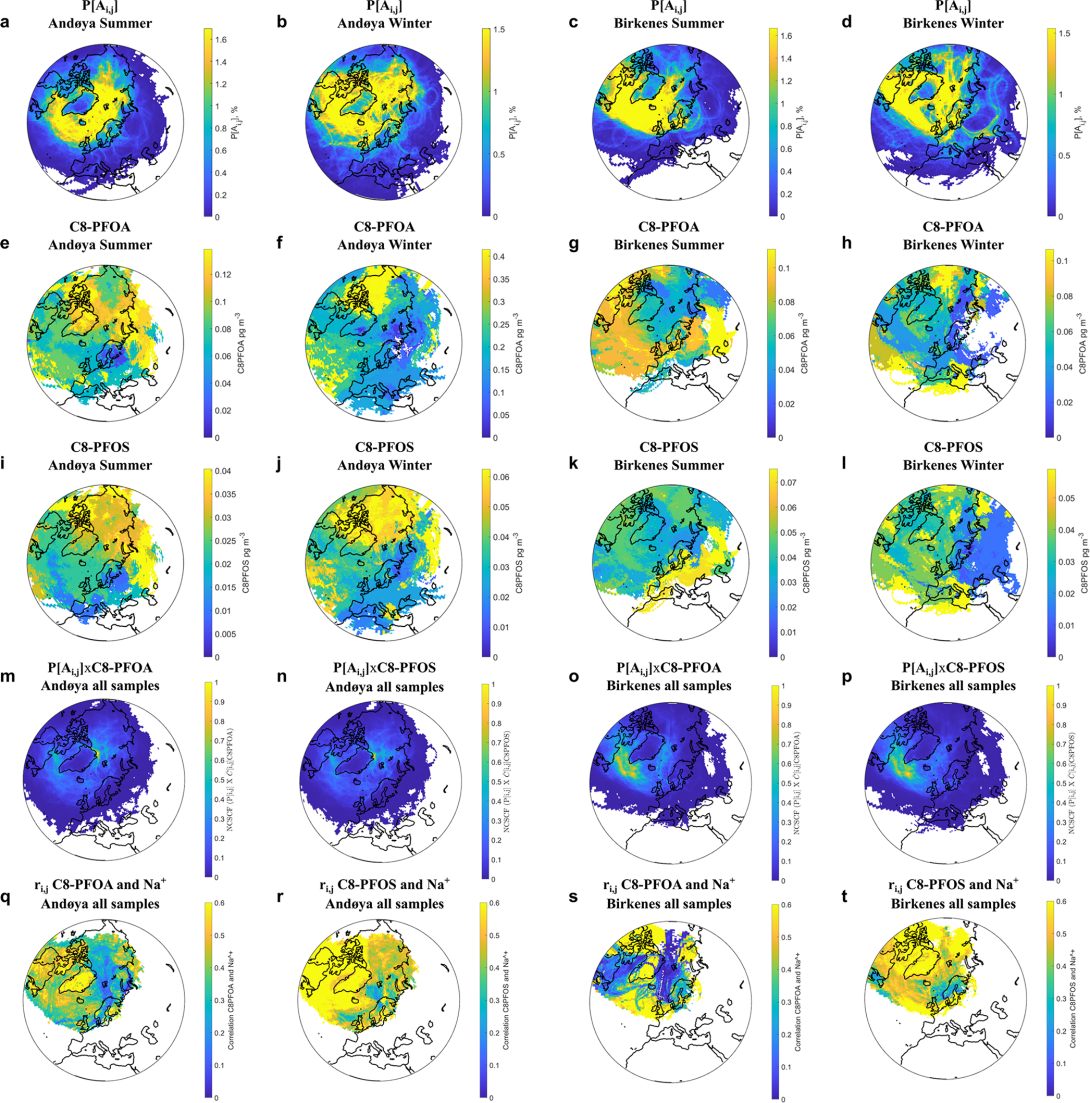
First row: air mass transport probability function (P[A_*i,j*_]) for the summer and winter samples at Andøya (a, b) and Birkenes (c, d). Second row: source attribution function (*C̅*_*i*__,*j*_) for PFOA in summer and winter samples at Andøya (e, f) and Birkenes (g, h). Third row: source attribution function for PFOS in summer and winter samples at Andøya (i, j) and Birkenes (k, l). Fourth row: *P*[*A*_*i*__,*j*_] × *C̅*_*i*__,*j*_ normalized to 1 for PFOA and PFOS in all samples from Andøya (m, n) and Birkenes (o, p). Fifth row: correlations (r_*i,j*_) between PFOA and Na^+^ and between PFOS and Na^+^ in all samples from Andøya (q, r) and Birkenes (s, t).

The results of the backward air mass trajectory analysis using the HYSPLIT4 model are presented in Figure 3. Both the Andøya and Birkenes samples are frequently influenced by air masses from the Arctic region and the North Atlantic Ocean as shown in the transport probability function plots (P[A_*i,j*_], the first row in Figure 3) for the two sampling sites. The trajectory pattern did not differ much between the summer and winter samples.

Potential source areas of PFAAs were tentatively identified based on the observed concentrations at the two sites using HYSPLIT4. The source areas of PFAAs revealed similar patterns so only the source attribution function plots (*C̅*_*i*__,*j*_) of PFOA and PFOS are shown in Figure 3 (the second and the third row) as an example. At Andøya, high PFAA concentrations were associated with air masses from the Arctic region and Eastern Europe. At Birkenes, PFCAs have a westerly origin from the North Atlantic in addition to Eastern Europe, especially in summer. PFSAs at Birkenes were generally associated with air masses from the Arctic region and the North Atlantic, but strong input from Europe/Eastern Europe may occur in summer. Such potential source area patterns may reflect transport of PFAAs via SSA from the Arctic and North Atlantic Ocean and the influence of precursor compounds from populated areas such as continental Europe. As production of C8-related fluorinated chemicals is still ongoing in Russia and China,^34^ emissions from these sources may also have affected the observed PFAA concentrations at the two sites. In order to estimate the accumulated source contribution to observed concentrations, the transport probability function and source attribution function can be combined (*P*[*A*_*i*__,*j*_] × *C̅*_*i*__,*j*_, the fourth row in Figure 3). This quantity, in the following normalized to 1, represents the overall contribution to the total amount of receptor-observed PFAAs and Na^+^, respectively, from each one of the grid-cells (defined under section 2.5). This method takes into account both the frequency of transport from each grid cell as well as the estimated relative strength of the source. The results, presented in the fourth row of Figure 3, suggest that air masses from the Arctic region and the North Atlantic appear to have contributed the most to the observed PFAA concentration at Andøya and Birkenes, respectively.

Plots of correlations per grid cell (*r*_*i,j*_, the fifth row in Figure 3) based on the observed PFAA and Na^+^ concentrations reveal higher *r*-values (0.4–0.6) in the Arctic region and North Atlantic than other areas, especially for PFOS (r and t in Figure 3) and PFHxS (Figure S6), suggesting PFAAs originated from these areas may be partly due to SSA emission. Plots for all PFAAs can be found in Figures S4–S6.

### 3.6 Comparison with the Previous Laboratory Study and Modeling Result

In a previous laboratory study,^18^ the enrichment factor of PFAAs in SSA was calculated as
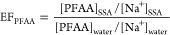
2After rearrangement of eq 2, the PFAA concentration contributed by SSA can be estimated by
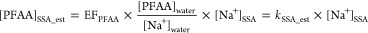
3where *k*_SSA_est_ (pg μg^–1^ Na^+^) represents the estimated amount of PFAA transferred to the atmosphere per μg of Na^+^. On the basis of laboratory-derived EFs and reported PFAA seawater concentrations from the literature and assuming a constant Na^+^ concentration of 13.8 g L^–1^ in water (35 g L^–1^ NaCl, same as the previous laboratory experiments^18^), *k*_SSA_ determined by the fitted lines for all samples from Andøya (Table S7) can be compared to *k*_SSA_est_ estimated using eq 3. Here the median concentrations of PFAAs in the Arctic Sea, North Atlantic, North Sea, and Baltic Sea provided in a recent review of PFAA concentration in seawater by Muir and Miaz^32^ were used for the estimation. The average EF of individual PFAAs in particles with a dry aerodynamic diameter < 10 μm from the previous laboratory study were used in the estimation.^18^ Details regarding the PFAA concentrations and EFs can be found in Table S8 and Table S9.

**Figure 4:**
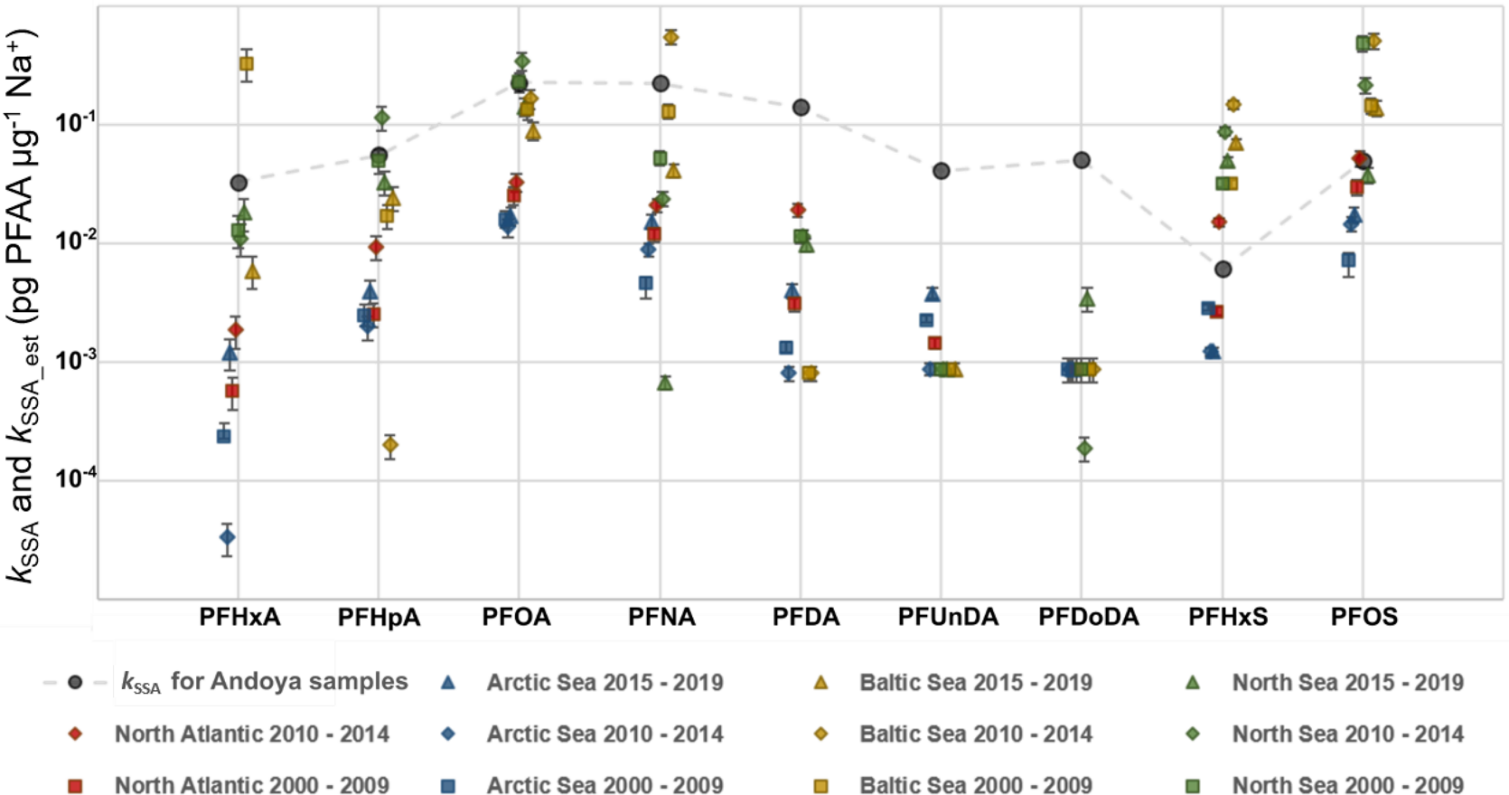
Comparison between the *k*_SSA_ determined by the linear fit (log10[PFAA] = log10(*k*_*SSA*_ × [Na^+^] + [PFAA]_other_) and the estimation, *k*_SSA_est_, based on previous laboratory results and median PFAA seawater concentrations from the literature. The error bar represents the standard deviation of *k*_SSA_est_, [PFAA]_seawater_ × (EF_Mean_ ± SD).

The comparison between *k*_SSA_ and *k*_SSA_est_ for Andøya are presented in Figure 4. In general, *k*_SSA_est_ followed a similar pattern to *k*_SSA_, but the differences between *k*_SSA_ and *k*_SSA_est_ can be 1–3 orders of magnitude. These estimates were greatly influenced by the PFAA seawater concentrations (i.e., the origin of the SSA). The relative standard deviation of the laboratory-derived EFs are between 8–30% (Table S9), but the median PFAA seawater concentrations in different regions vary over orders of magnitude. For example, the median seawater concentrations of PFOA between 2010 and 2014 are 43, 103, 520, and 1090 pg m^–3^ for samples from the Arctic Sea, North Atlantic, Baltic Sea, and North Sea, respectively.^32^ Such large differences highlight the importance of accurate information on the geospatial variation of PFAA seawater concentrations when evaluating the contribution of SSA. It should be noted that for PFCAs, *k*_SSA_est_ is at least 2 times lower than *k*_SSA_ in most cases, especially when the median PFAA seawater concentrations for the time period of 2015–2019 were used. The causes of the underestimation are unclear since the enrichment process in the field is much more complicated than in controlled laboratory experiments. For example, other ions in seawater such as Mg^2+^ also contribute to SSA emission while only NaCl was used in the laboratory study; the presence of organic matter in seawater may influence PFAAs emission by SSA; wind speed at the sea surface may influence the amount and size of SSA particles emitted; and the SSA size distribution at the sampling site can be very different from freshly emitted SSA, etc. As such, further research on the PFAAs enrichment mechanism in SSA is required to reduce the uncertainty.

On the basis of modeled annual SSA production and *k*_SSA_ determined from the fitted lines for all samples from Andøya (Table S7), the global annual fluxes of PFOA and PFOS from the ocean to the atmosphere via SSA emission can be estimated as
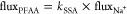
4*k*_SSA_ of the Andøya samples was used since the sampling site at Andøya is ∼1.3 km to open water so the SSA size distribution may be more similar to nascent SSA above the sea surface than at Birkenes. For SSA annual production (flux_Na+_), Textor et al. calculated 12 estimates of SSA annual production using 12 chemical transport and general circulation models.^44^ The first and third quartile and the median values of these 12 estimates were used as the low, high, and median scenarios. These estimates of SSA annual production have recently been updated,^45^ but the values reported by Textor et al. were still used in the present study in order to compare with the modeled PFOA and PFOS emission via SSA by Johansson et al.^15^ The estimated global annual fluxes of PFOA and PFOS and comparison with Johansson et al.^15^ are shown in Table S10. Estimates of PFOA and PFOS fluxes using the updated SSA annual production are also included in Table S10 for reference.

For PFCAs, the modeling results from the three scenarios by Johansson et al. (23, 122, and 506 tonnes yr^–1^ for the low, median, and high scenario, respectively)^15^ are all below the estimates based on the field measurements (258, 442, and 686 tonnes yr^–1^ for the low, median, and high scenario, respectively). Similarly, in the previous comparison with the laboratory result, *k*_SSA_est_ of PFCAs were also lower than *k*_SSA_ in most cases (Figure 4). This suggests that using lab-derived EFs may underestimate the contribution of SSA to PFCAs in air. The estimated annual flux of PFOS in the low scenario (56 tonnes yr^–1^) is close to the modeling result of Johansson et al. (42 tonnes yr^–1^), while the median (96 tonnes yr^–1^) and high scenarios (149 tonnes yr^–1^) are lower than the previous modeling results (183 and 801 tonnes yr^–1^ for the median and high scenarios, respectively).^15^ As mentioned before, PFAA seawater concentrations have a large influence on estimates of the amount of PFAA transferred to air via SSA emission (*k*_SSA_est_) based on laboratory-derived EFs. So the PFAA seawater concentrations that are used as input in the modeling may be one cause of the differences between the modeling results and the estimated annual fluxes. In addition, artificial seawater without organic matter was used to derive the EFs in the lab. But the composition of real seawater is very complex and may have influence on the enrichment process,^17,19^ so the EFs measured in the lab may differ from the field. It should also be noted that the model used the EF of three modes as input (0.095, 0.6, and 1.5 μm), while the estimate in the current study was based on the field measurement of bulk aerosol samples. The EFs generally increase with decreasing particle size.^15,18^ For example, the laboratory-derived average EF of PFOS was 9.6 ± 1.4 × 10^3^ for particles < 10 μm but increased to 2.9 ± 0.5 × 10^4^ when only particles < 1.5 μm were considered (Table S8).

The observed significant correlation between PFAAs and Na^+^ in the samples from Andøya and Birkenes suggest that SSA can be an important source of atmospheric PFAAs in coastal areas. Further, since SSA particles can travel significant distances in the atmosphere (e.g., an SSA particle with *r*_80_ = 5 μm can spend upward of 10 h in the atmosphere and travel more than 300 km), our results suggest that PFAAs transport via SSA may impact large areas of inland Europe and other continents in addition to coastal areas. However, it is still challenging to evaluate the contribution from SSA on the global scale. Further laboratory experiments focusing on the enrichment mechanisms and more field evidence from various coastal locations are required before this process is well-parametrized for inclusion in global models.

## Supporting Information Available

(S1) Sample extraction and instrumental analysis; (S2) QA/QC; (S3) backward air mass trajectory analysis using HYSPLIT4 references; (Figure S1) locations of the sampling sites; (Figure S2) correlations between individual PFAAs in air samples at the two locations; (Figure S3) correlations between Na^+^ and Mg^2+^ ions at Andøya and Birkenes; (Figure S4) transport probability plots (P[A_ij_]) for summer, winter, and all samples from each site; (Figure S5) source attribution function plots (*C*_ij_) for the summer and winter samples from each site; (Figure S6) P[A_ij_] × C_ij_ plots for all samples from each sampling site; (Table S1) target compounds; (Table S2) standards and reagents; (Table S3) start date, end date, sampling duration, and total volume of each sample collected at Andøya and Birkenes; (Table S4) laboratory blanks, field blanks, MDLs, IS recovery, and result of spike recovery test; (Table S5) results of Pearson correlation and orthogonal linear regressions between PFAA concentrations and Na^+^ concentration in the samples; (Table S6) detection frequencies and concentration ranges of the analytes; (Table S7) results of Pearson correlation between PFAA concentrations and Na^+^ concentration in the summer and winter samples; (Table S8) enrichment factors from the previous laboratory study using a SSA chamber; (Table S9) medians and sample numbers for all coastal and open ocean sites from Muir and Miaz; and (Table S10) estimated annual global PFOA and PFOS emissions via SSA (PDF).

## References

[ref1] Ahrens L. Polyfluoroalkyl Compounds in the Aquatic Environment: A Review of Their Occurrence and Fate. J. Environ. Monit. 2011, 13 (1), 20–31. 10.1039/C0EM00373E. 21031178

[ref2] Ahrens L.; Gerwinski W.; Theobald N.; Ebinghaus R. Sources of Polyfluoroalkyl Compounds in the North Sea, Baltic Sea and Norwegian Sea: Evidence from Their Spatial Distribution in Surface Water. Mar. Pollut. Bull. 2010, 60 (2), 255–260. 10.1016/j.marpolbul.2009.09.013. 19818459

[ref3] Dreyer A.; Weinberg I.; Temme C.; Ebinghaus R. Polyfluorinated Compounds in the Atmosphere of the Atlantic and Southern Oceans: Evidence for a Global Distribution. Environ. Sci. Technol. 2009, 43 (17), 6507–6514. 10.1021/es9010465. 19764209

[ref4] Giesy J. P.; Kannan K. Global Distribution of Perfluorooctane Sulfonate in Wildlife. Environ. Sci. Technol. 2001, 35 (7), 1339–1342. 10.1021/es001834k. 11348064

[ref5] Haug L. S.; Thomsen C.; Becher G. Time Trends and the Influence of Age and Gender on Serum Concentrations of Perfluorinated Compounds in Archived Human Samples. Environ. Sci. Technol. 2009, 43 (6), 2131–2136. 10.1021/es802827u. 19368225

[ref6] Yamashita N.; Kannan K.; Taniyasu S.; Horii Y.; Petrick G.; Gamo T. A Global Survey of Perfluorinated Acids in Oceans. Mar. Pollut. Bull. 2005, 51 (8), 658–668. 10.1016/j.marpolbul.2005.04.026. 15913661

[ref7] Casal P.; Zhang Y.; Martin J. W.; Pizarro M.; Jiménez B.; Dachs J. Role of Snow Deposition of Perfluoroalkylated Substances at Coastal Livingston Island (Maritime Antarctica). Environ. Sci. Technol. 2017, 51 (15), 8460–8470. 10.1021/acs.est.7b02521. 28665121

[ref8] Zheng H.; Wang F.; Zhao Z.; Ma Y.; Yang H.; Lu Z.; Cai M.; Cai M. Distribution Profiles of Per- and Poly Fluoroalkyl Substances (PFASs) and Their Re-Regulation by Ocean Currents in the East and South China Sea. Mar. Pollut. Bull. 2017, 125 (1), 481–486. 10.1016/j.marpolbul.2017.08.009. 28800911

[ref9] Casas G.; Martínez-Varela A.; Roscales J. L.; Vila-Costa M.; Dachs J.; Jiménez B. Enrichment of Perfluoroalkyl Substances in the Sea-Surface Microlayer and Sea-Spray Aerosols in the Southern Ocean. Environ. Pollut. 2020, 267, 115512 10.1016/j.envpol.2020.115512. 32892018

[ref10] Prevedouros K.; Cousins I. T.; Buck R. C.; Korzeniowski S. H. Sources, Fate and Transport of Perfluorocarboxylates. Environ. Sci. Technol. 2006, 40 (1), 32–44. 10.1021/es0512475. 16433330

[ref11] Armitage J.; Cousins I. T.; Buck R. C.; Prevedouros K.; Russell M. H.; MacLeod M.; Korzeniowski S. H. Modeling Global-Scale Fate and Transport of Perfluorooctanoate Emitted from Direct Sources. Environ. Sci. Technol. 2006, 40 (22), 6969–6975. 10.1021/es0614870. 17154003

[ref12] Armitage J. M.; MacLeod M.; Cousins I. T. Modeling the Global Fate and Transport of Perfluorooctanoic Acid (PFOA) and Perfluorooctanoate (PFO) Emitted from Direct Sources Using a Multispecies Mass Balance Model. Environ. Sci. Technol. 2009, 43 (4), 1134–1140. 10.1021/es802900n. 19320170

[ref13] Thackray C. P.; Selin N. E.; Young C. J. A Global Atmospheric Chemistry Model for the Fate and Transport of PFCAs and Their Precursors. Environ. Sci. Process. Impacts 2020, 22 (2), 285–293. 10.1039/C9EM00326F. 31942888 PMC7050637

[ref14] Young C. J.; Mabury S. A. Atmospheric Perfluorinated Acid Precursors: Chemistry, Occurrence, and Impacts. In Reviews of Environmental Contamination and Toxicology Vol. 208: Perfluorinated alkylated substances; De Voogt P., Ed.; Reviews of Environmental Contamination and Toxicology; Springer: New York, NY, 2010; pp 1–109, 10.1007/978-1-4419-6880-7_1. 20811862

[ref15] Johansson J. H.; Salter M. E.; Acosta Navarro J. C.; Leck C.; Nilsson E. D.; Cousins I. T. Global Transport of Perfluoroalkyl Acids via Sea Spray Aerosol. Environ. Sci. Process. Impacts 2019, 21 (4), 635–649. 10.1039/C8EM00525G. 30888351

[ref16] Reth M.; Berger U.; Broman D.; Cousins I. T.; Nilsson E. D.; McLachlan M. S. Water-to-Air Transfer of Perfluorinated Carboxylates and Sulfonates in a Sea Spray Simulator. Environ. Chem. 2011, 8 (4), 381–388. 10.1071/EN11007.

[ref17] de Leeuw G.; Andreas E. L; Anguelova M. D.; Fairall C. W.; Lewis E. R.; O'Dowd C.; Schulz M.; Schwartz S. E. Production Flux of Sea Spray Aerosol. Rev. Geophys. 2011, 49 (2), RG2001 10.1029/2010RG000349.

[ref18] Sha B.; Johansson J. H.; Benskin J. P.; Cousins I. T.; Salter M. E. Influence of Water Concentrations of Perfluoroalkyl Acids (PFAAs) on Their Size-Resolved Enrichment in Nascent Sea Spray Aerosols. Environ. Sci. Technol. 2021, 55, 9489 10.1021/acs.est.0c03804. 32859129 PMC8296677

[ref19] Lewis E. R.; Schwartz S. E. Sea Salt Aerosol Production: Mechanisms, Methods, Measurements and Models; Geophysical Monograph Series; AGU: Washington D.C., 2004; Vol. 152.

[ref20] McMurdo C. J.; Ellis D. A.; Webster E.; Butler J.; Christensen R. D.; Reid L. K. Aerosol Enrichment of the Surfactant PFO and Mediation of the Water-Air Transport of Gaseous PFOA. Environ. Sci. Technol. 2008, 42 (11), 3969–3974. 10.1021/es7032026. 18589953

[ref21] Wong F.; Shoeib M.; Katsoyiannis A.; Eckhardt S.; Stohl A.; Bohlin-Nizzetto P.; Li H.; Fellin P.; Su Y.; Hung H. Assessing Temporal Trends and Source Regions of Per- and Polyfluoroalkyl Substances (PFASs) in Air under the Arctic Monitoring and Assessment Programme (AMAP). Atmos. Environ. 2018, 172, 65–73. 10.1016/j.atmosenv.2017.10.028.

[ref22] Kwok K. Y.; Yamazaki E.; Yamashita N.; Taniyasu S.; Murphy M. B.; Horii Y.; Petrick G.; Kallerborn R.; Kannan K.; Murano K.; Lam P. K. S. Transport of Perfluoroalkyl Substances (PFAS) from an Arctic Glacier to Downstream Locations: Implications for Sources. Sci. Total Environ. 2013, 447, 46–55. 10.1016/j.scitotenv.2012.10.091. 23376515

[ref23] MacInnis J. J.; French K.; Muir D. C. G.; Spencer C.; Criscitiello A.; De Silva A. O.; Young C. J. Emerging Investigator Series: A 14-Year Depositional Ice Record of Perfluoroalkyl Substances in the High Arctic. Environ. Sci. Process. Impacts 2017, 19 (1), 22–30. 10.1039/C6EM00593D. 28092384

[ref24] Young C. J.; Furdui V. I.; Franklin J.; Koerner R. M.; Muir D. C. G.; Mabury S. A. Perfluorinated Acids in Arctic Snow: New Evidence for Atmospheric Formation. Environ. Sci. Technol. 2007, 41 (10), 3455–3461. 10.1021/es0626234. 17547163

[ref25] Pickard H. M.; Criscitiello A. S.; Spencer C.; Sharp M. J.; Muir D. C. G.; De Silva A. O.; Young C. J. Continuous Non-Marine Inputs of per- and Polyfluoroalkyl Substances to the High Arctic: A Multi-Decadal Temporal Record. Atmos. Chem. Phys. 2018, 18 (7), 5045–5058. 10.5194/acp-18-5045-2018.

[ref26] Wang Z.; Cousins I. T.; Scheringer M.; Buck R. C.; Hungerbühler K. Global Emission Inventories for C4-C14 Perfluoroalkyl Carboxylic Acid (PFCA) Homologues from 1951 to 2030, Part II: The Remaining Pieces of the Puzzle. Environ. Int. 2014, 69, 166–176. 10.1016/j.envint.2014.04.006. 24861268

[ref27] Wang Z.; Boucher J. M.; Scheringer M.; Cousins I. T.; Hungerbühler K. Toward a Comprehensive Global Emission Inventory of C4-C10 Perfluoroalkanesulfonic Acids (PFSAs) and Related Precursors: Focus on the Life Cycle of C8-Based Products and Ongoing Industrial Transition. Environ. Sci. Technol. 2017, 51 (8), 4482–4493. 10.1021/acs.est.6b06191. 28323424

[ref28] Benskin J. P.; Ikonomou M. G.; Woudneh M. B.; Cosgrove J. R. Rapid Characterization of Perfluoralkyl Carboxylate, Sulfonate, and Sulfonamide Isomers by High-Performance Liquid Chromatography-Tandem Mass Spectrometry. J. Chromatogr. A 2012, 1247, 165–170. 10.1016/j.chroma.2012.05.077. 22695697

[ref29] Draxler R. R.; Hess G. D. An Overview of the HYSPLIT_4 Modelling System for Trajectories, Dispersion, and Deposition. Aust. Meteorol. Mag. 1998, 47, 295–308.

[ref30] Bohlin-Nizzetto P.; Aas W.; Nikiforov V. Monitoring of Environmental Contaminants in Air and Precipitation, Annual Report 2018; NILU, 2019.

[ref31] Bohlin-Nizzetto P.; Aas W.; Nikiforov V. Monitoring of Environmental Contaminants in Air and Precipitation Annual Report 2019; NILU, 2020.

[ref32] Muir D.; Miaz L. T. Spatial and Temporal Trends of Perfluoroalkyl Substances in Global Ocean and Coastal Waters. Environ. Sci. Technol. 2021, 55, 9527 10.1021/acs.est.0c08035. 33646763

[ref33] Hoffman G. L.; Duce R. A. Consideration of the Chemical Fractionation of Alkali and Alkaline Earth Metals in the Hawaiian Marine Atmosphere. J. Geophys. Res. 1972, 77 (27), 5161–5169. 10.1029/JC077i027p05161.

[ref34] Muir D.; Bossi R.; Carlsson P.; Evans M.; De Silva A.; Halsall C.; Rauert C.; Herzke D.; Hung H.; Letcher R.; Rigét F.; Roos A. Levels and Trends of Poly- and Perfluoroalkyl Substances in the Arctic Environment - An Update. Emerg. Contam. 2019, 5, 240–271. 10.1016/j.emcon.2019.06.002.

[ref35] Joerss H.; Apel C.; Ebinghaus R. Emerging Per- and Polyfluoroalkyl Substances (PFASs) in Surface Water and Sediment of the North and Baltic Seas. Sci. Total Environ. 2019, 686, 360–369. 10.1016/j.scitotenv.2019.05.363. 31181522

[ref36] Joerss H.; Xie Z.; Wagner C. C.; von Appen W.-J.; Sunderland E. M.; Ebinghaus R. Transport of Legacy Perfluoroalkyl Substances and the Replacement Compound HFPO-DA through the Atlantic Gateway to the Arctic Ocean—Is the Arctic a Sink or a Source?. Environ. Sci. Technol. 2020, 54 (16), 9958–9967. 10.1021/acs.est.0c00228. 32806910 PMC7733389

[ref37] Yeung L. W. Y.; Dassuncao C.; Mabury S.; Sunderland E. M.; Zhang X.; Lohmann R. Vertical Profiles, Sources, and Transport of PFASs in the Arctic Ocean. Environ. Sci. Technol. 2017, 51 (12), 6735–6744. 10.1021/acs.est.7b00788. 28513149

[ref38] Li L.; Zheng H.; Wang T.; Cai M.; Wang P. Perfluoroalkyl Acids in Surface Seawater from the North Pacific to the Arctic Ocean: Contamination, Distribution and Transportation. Environ. Pollut. 2018, 238, 168–176. 10.1016/j.envpol.2018.03.018. 29554564

[ref39] Benskin J. P.; Ahrens L.; Muir D. C. G.; Scott B. F.; Spencer C.; Rosenberg B.; Tomy G.; Kylin H.; Lohmann R.; Martin J. W. Manufacturing Origin of Perfluorooctanoate (PFOA) in Atlantic and Canadian Arctic Seawater. Environ. Sci. Technol. 2012, 46 (2), 677–685. 10.1021/es202958p. 22128769

[ref40] Herzke D.; Olsson E.; Posner S. Perfluoroalkyl and Polyfluoroalkyl Substances (PFASs) in Consumer Products in Norway - A Pilot Study. Chemosphere 2012, 88 (8), 980–987. 10.1016/j.chemosphere.2012.03.035. 22483730

[ref41] Boucher J. M.; Cousins I. T.; Scheringer M.; Hungerbühler K.; Wang Z. Toward a Comprehensive Global Emission Inventory of C4-C10 Perfluoroalkanesulfonic Acids (PFSAs) and Related Precursors: Focus on the Life Cycle of C6- and C10-Based Products. Environ. Sci. Technol. Lett. 2019, 6 (1), 1–7. 10.1021/acs.estlett.8b00531. 28323424

[ref42] Hurley M. D.; Wallington T. J.; Sulbaek Andersen M. P.; Ellis D. A.; Martin J. W.; Mabury S. A. Atmospheric Chemistry of Fluorinated Alcohols: Reaction with Cl Atoms and OH Radicals and Atmospheric Lifetimes. J. Phys. Chem. A 2004, 108 (11), 1973–1979. 10.1021/jp0373088.

[ref43] Wallington T. J.; Hurley M. D.; Xia J.; Wuebbles D. J.; Sillman S.; Ito A.; Penner J. E.; Ellis D. A.; Martin J.; Mabury S. A.; Nielsen O. J.; Sulbaek Andersen M. P. Formation of C7F15COOH (PFOA) and Other Perfluorocarboxylic Acids during the Atmospheric Oxidation of 8:2 Fluorotelomer Alcohol. Environ. Sci. Technol. 2006, 40 (3), 924–930. 10.1021/es051858x. 16509338

[ref44] Textor C.; Schulz M.; Guibert S.; Kinne S.; Balkanski Y.; Bauer S.; Berntsen T.; Berglen T.; Boucher O.; Chin M.; Dentener F.; Diehl T.; Easter R.; Feichter H.; Fillmore D.; Ghan S.; Ginoux P.; Gong S.; Grini A.; Hendricks J.; Horowitz L.; Huang P.; Isaksen I.; Iversen I.; Kloster S.; Koch D.; Kirkevåg A.; Kristjansson J. E.; Krol M.; Lauer A.; Lamarque J. F.; Liu X.; Montanaro V.; Myhre G.; Penner J.; Pitari G.; Reddy S.; Seland Å.; Stier P.; Takemura T.; Tie X. Analysis and Quantification of the Diversities of Aerosol Life Cycles within AeroCom. Atmos. Chem. Phys. 2006, 6 (7), 1777–1813. 10.5194/acp-6-1777-2006.

[ref45] Gliß J.; Mortier A.; Schulz M.; Andrews E.; Balkanski Y.; Bauer S. E.; Benedictow A. M. K.; Bian H.; Checa-Garcia R.; Chin M.; Ginoux P.; Griesfeller J. J.; Heckel A.; Kipling Z.; Kirkevåg A.; Kokkola H.; Laj P.; Le Sager P.; Lund M. T.; Lund Myhre C.; Matsui H.; Myhre G.; Neubauer D.; van Noije T.; North P.; Olivié D. J. L.; Rémy S.; Sogacheva L.; Takemura T.; Tsigaridis K.; Tsyro S. G. AeroCom Phase III Multi-Model Evaluation of the Aerosol Life Cycle and Optical Properties Using Ground- and Space-Based Remote Sensing as Well as Surface in Situ Observations. Atmos. Chem. Phys. 2021, 21 (1), 87–128. 10.5194/acp-21-87-2021.

