# Supplementary material for: Sea Spray Aerosol (SSA) as a Source of Perfluoroalkyl Acids (PFAAs) to the Atmosphere: Field Evidence from Long-Term Air Monitoring
Source: Environ Sci Technol. 2021 Dec 15;56(1):228–38. doi: 10.1021/acs.est.1c04277 (PMC8733926; doi:10.1021/acs.est.1c04277)
Supplement: Supplementary file 1 — es1c04277_si_001.pdf [file es1c04277_si_001.pdf]

# Supporting information

## Importance of sea spray aerosol (SSA) as a source of perfluoroalkyl acids (PFAAs) to the atmosphere: field evidence from long-term air monitoring

Bo Sha *[a], Jana H. Johansson [a], Peter Tunved [a b], Pernilla Bohlin-Nizzetto [c], Ian T. Cousins [a], Matthew E. Salter [a b]

[a] Department of Environmental Science, Stockholm University, SE-106 91, Stockholm, Sweden.

[b] Bolin Centre for Climate Research, SE-106 91, Stockholm, Sweden.

[c] NILU - Norwegian Institute for Air Research, P.O. Box 100, 2027 Kjeller, Norway.

Total number of pages: 23

Total number of figures: 6

Total number of tables: 10

# Table of contents

S1. Sample extraction and instrumental analysis S5
S2. QA/QC S5
S3. Backward air mass trajectory analysis using HYSPLIT4 S5
References S23

# List of figures

Figure S1. Locations of the sampling sties. S7

Figure S2. Correlations between individual PFAAs in air samples at the two locations. S7

Figure S3. Correlations between $Na^+$ and $Mg^{2+}$ ions at Andøya and Birkenes. S8

Figure S4 Transport probability plots ($P[A_{ij}]$) for summer, winter and all samples from each site. S9

Figure S5 Source attribution function plots ($C_{ij}$) for the summer and winter samples from each site. S10

Figure S6 $P[A_{ij}]xC_{ij}$ plots for all samples from each sampling site. S11

## List of tables

Table S1. Target compounds. S12

Table S2. Standards and reagents. S13

Table S3. The start date, end date, sampling duration and total volume of each sample collected at Andøya and Birkenes. S14

Table S4. Laboratory blanks, field blanks, MDLs, IS recovery and result of spike recovery test. S16

Table S5. Results of Pearson correlation and orthogonal linear regressions between PFAA concentrations and $Na^+$ concentration in the samples. S17

Table S6. Detection frequencies and concentration ranges of the analytes. S18

Table S7. Results of Pearson correlation between PFAA concentrations and $Na^+$ concentration in the summer and winter samples. S19

Table S8. Enrichment factors from the previous laboratory study using a SSA chamber. S20

Table S9. Medians and sample numbers for all coastal and open ocean sites from Muir and Miaz. S21

Table S10. Estimated annual global PFOA and PFOS emissions via SSA. S22

## S1. Sample extraction and instrumental analysis

Prior to extraction, a small hole (φ=13 mm) was punched in each QFF. The punch was stored for sodium ($Na^+$) and magnesium ($Mg^{2+}$) analysis. The rest of the filter was used for PFAA analysis. It was first spiked with mass-labelled internal standards (IS) and then extracted by sonicating three times in 25 mL methanol (MeOH) for 30 min. The extract was evaporated to dryness and reconstituted in 50% MeOH and 50% 4mM ammonium acetate in MilliQ water to a final volume of 300 µL. Recovery standards (RS) were added prior to PFAA analysis. The small punch taken from each QFF was sonicated in 3 mL MilliQ water for 15 min and then centrifuged at 4000 rpm for 10 min. The supernatant was collected for ion analysis.

PFAAs were analyzed on an Acquity ultra-performance liquid chromatography system coupled to a Xevo TQ-S tandem mass spectrometer (UPLC/MS/MS; Waters Corp.) based on a previously published method.[1] Briefly, 50 µL of the final extract was injected on an Ascentis Express F5 PFP Column (2.7 µm, 10 cm × 2.1 mm, Sigma-Aldrich) equipped with an Ascentis Express F5 PFP guard column (2.7 µm, 5.0 mm × 2.1 mm). The columns were both maintained at 30°C. The mobile phase consisted of A) 2 mM ammonium formate and 2 mM formic acid in MilliQ water; and B) MeOH. The flow rate of the mobile phase was 0.25 mL/min and the mass spectrometer was operated in negative electrospray ionization mode. Gradient conditions were: 90% B for 1 min, 40% B by 3 min, 12% B by 14 min, 0% B by 14.5 min, 0% B by 14.5 min, then 90% B and equilibrated for 6.5 min. Any background contamination originating from the instrument and mobile phase was delayed by using a “PFC isolator column” from Waters “PFC kit" placed before the injector.

## S2. QA/QC

For PFAAs, the method detection limits (MDLs) were determined by the mean value plus 3 times the standard deviation of the laboratory blanks (mean+3×SD). PFHxA, PFOA, and PFDoDA concentrations in more than 30% of the field blanks were above the MDLs, while all the other PFAAs were only occasionally detected in one or two field blanks. The reported PFHxA, PFOA, and PFDoDA concentrations were corrected by the mean values of the field blanks. $Na^+$ and $Mg^{2+}$ ions were not detected in the blanks and their MDLs were reported as 3 times the signal-to-noise level (S/N).

The mean recoveries of the mass-labelled internal standards of PFAAs were between 20–70%. As part of the quality assurance, the native compound in a sample was marked as “not detected” and excluded from the following analysis if the recovery of the corresponding IS was <15%.

Spike-recovery tests for PFAAs were performed at two levels, 30 pg (*n*=3) and 300 pg (*n*=3), of individual PFAAs per QFF. Pre-baked QFFs were spiked with PFAA mixtures and then treated as real samples. The recoveries (mean±SD) of the native PFAAs were between 79±12% and 102±26%.

## S3. Backward air mass trajectory analysis using HYSPLIT4

HYSPLIT4, configured for the ensemble method, was used for backward air mass trajectory analysis. It is estimated that, for SSA with a radius at 80% relative humidity ≤3 µm, the mean atmospheric residence time is ~3 days (to within a factor of two or three)[2], so the duration of 10 days is used for the backward air mass trajectory analysis. The ensemble method offsets the meteorological grid by a single grid point in latitude, longitude and altitude, producing 27 back-trajectories for all possible offsets in the horizontal and vertical. This approach was used to account for uncertainties in the gridded meteorological data. Through the sampling period, individual hourly timestamps for each sample were recorded. For each one of these hourly timestamps an ensemble trajectory run was performed. Given that the sampling time typically is 48 h, this

results in 48 ensemble trajectories giving a total of 1296 trajectories for each sample. These data were subsequently analyzed with respect to air-mass origin by mapping the individual trajectory endpoints on a polar grid centered around either of the two receptors Andøya or Birkenes. The grid, centered on each station, covers latitudes 0-90 degrees latitude (after pole-transformation of the grid) and -180 to 180 degrees longitude with a total number of 180×180 grid cells.

The transport probability function plot ($P[A_{i,j}]$) shows the dominant transport paths at Andøya and Birkenes for all samples, the summer samples and winter samples, respectively. The transport probability function is given by:

$$P[A_{i,j}] = \frac{n_{i,j}}{N}$$

where $n_{i,}$ is the number of trajectories crossing the grid cell (*i*,*j*) and N is the total number of trajectories in the dataset (summer, winter or all samples at the two locations). This type of plot represents the probability of air masses crossing a certain grid cell before reaching the sampling sites.

The source attribution function plot ($C_{i,j}$) relates the transport over grid $n_{i,j}$ to the observed concentration at the receptor (Andøya and Birkenes). Mathematically, the average concentration in a grid cell (*i*,*j*) is calculated as:

$$\bar{C}_{i,j} = \frac{\sum c_{i,j} \times n_{i,j}}{\sum n_{i,j}}$$

where $\bar{C}_{i,j}$ is the average observed concentration at the receptor after crossing grid (*i*,*j*), and $c_{i,j}$ is the individual observed concentration linked to each grid cell. Hence, the figures represent the average observed concentrations of PFAAs and $Na^+$ if the trajectory has spent time in the grid (*i*,*j*). In other words, this type of plot represents the “footprint” of PFAAs and $Na^+$ in each grid cell. In order to evaluate the relative contribution of each source area to the observed concentrations at the sampling sites, the source attribution function has to be balanced by the transport probability function, i.e. $P[A_{i,j}] \times \bar{C}_{i,j}$.

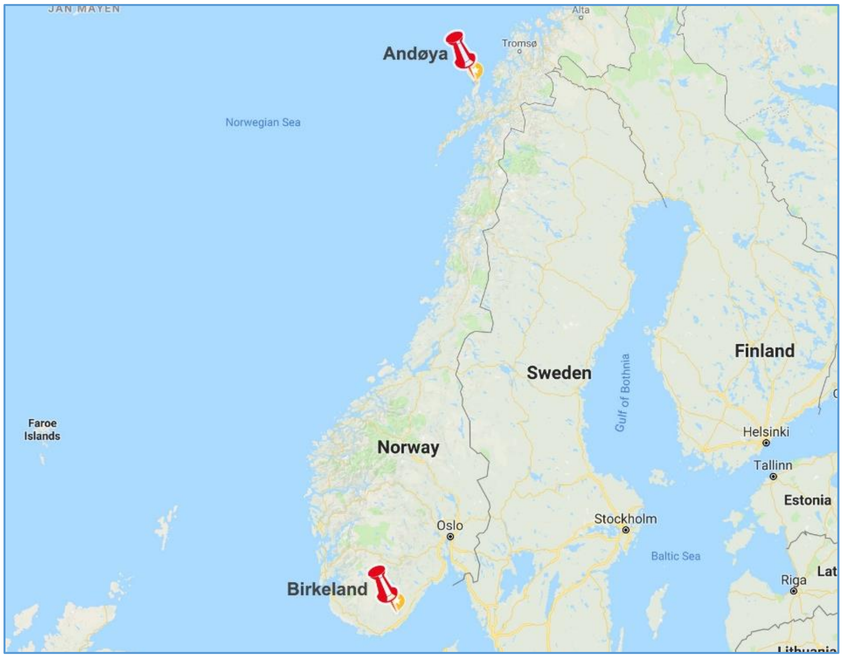


**Figure S1. Locations of the sampling sties.**

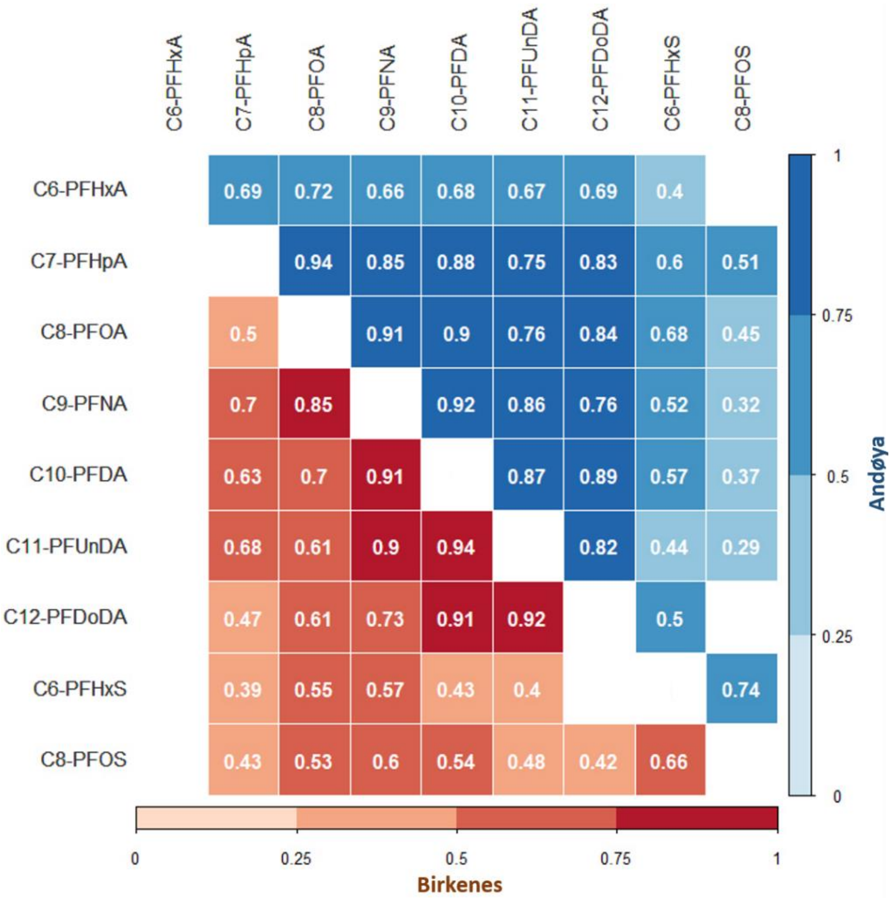


**Figure S2. Correlations between individual PFAAs in air samples at the two locations. The shading of each square and the numbers inside each square indicate the strength (*r*-value) of the Pearson correlation. Only correlations with *p*<0.05 are shown.**

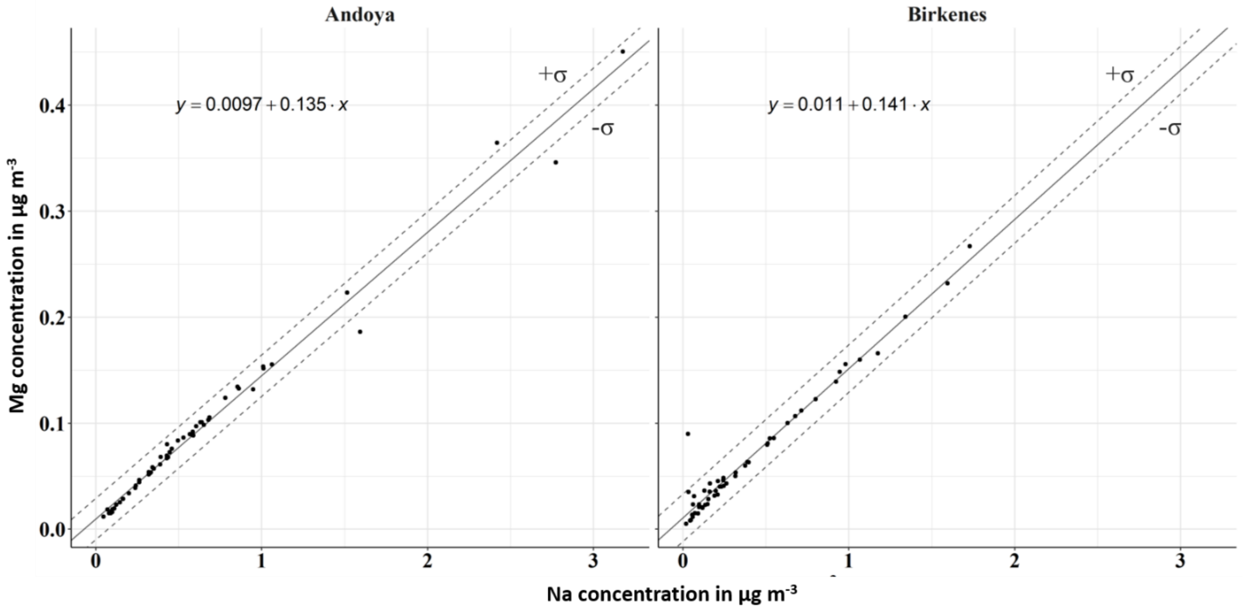


**Figure S3. Correlations between $Na^{+}$ and $Mg^{2+}$ ions at Andøya and Birkenes. The solid lines are fitted by orthogonal regression and the dashed lines represent ±σ.**

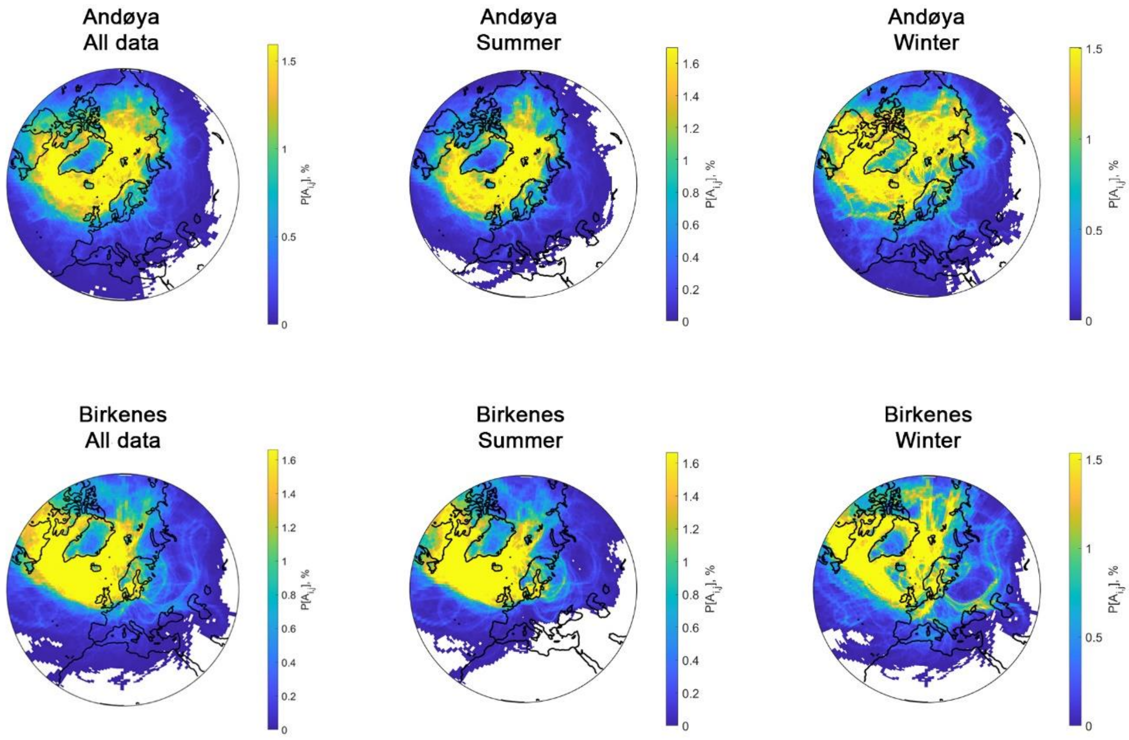


**Figure S4 Transport probability plots (P[Aij]) for summer, winter and all samples from each site.**

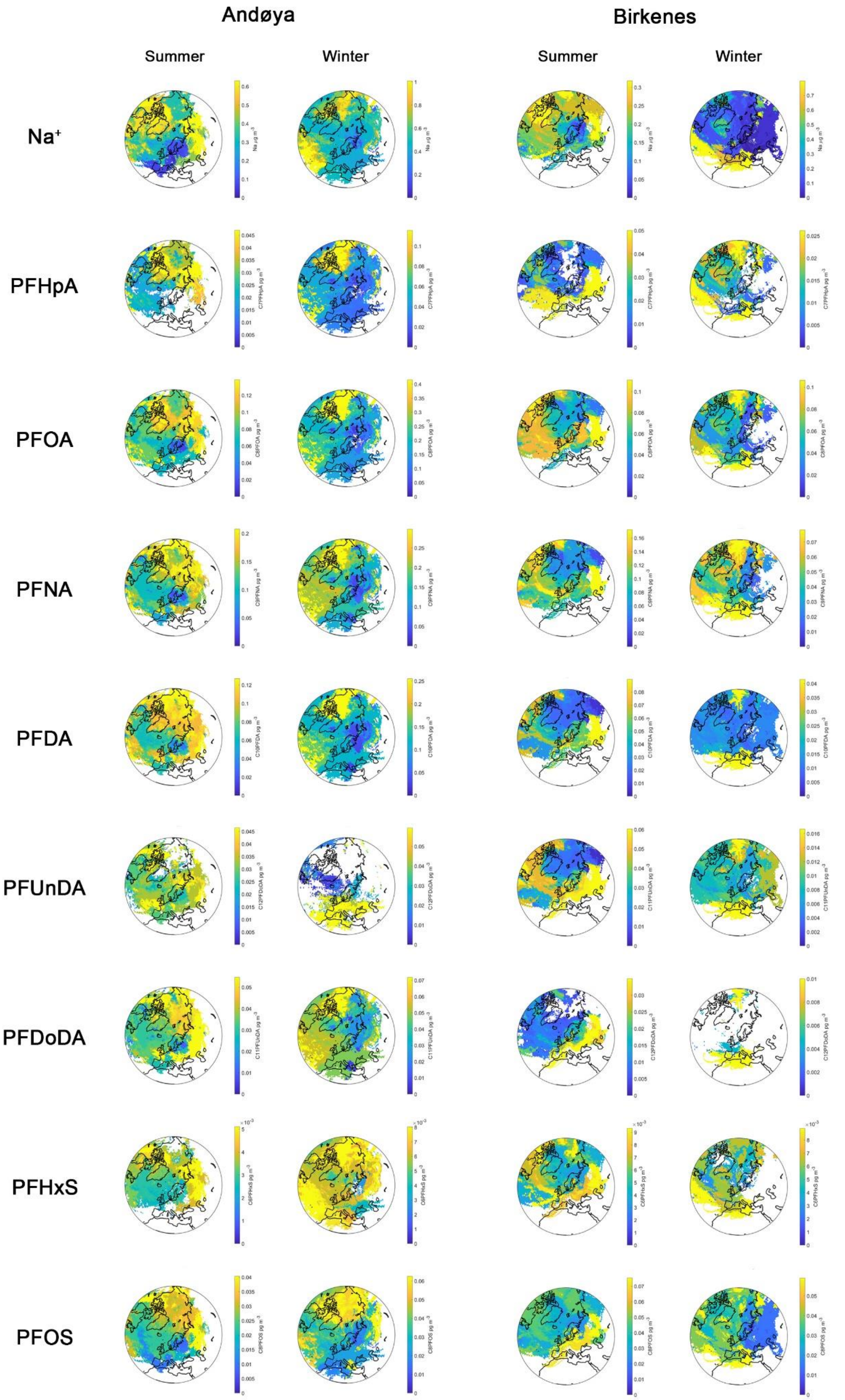


**Figure S5 Source attribution function plots (Cij) for the summer and winter samples from each site.**

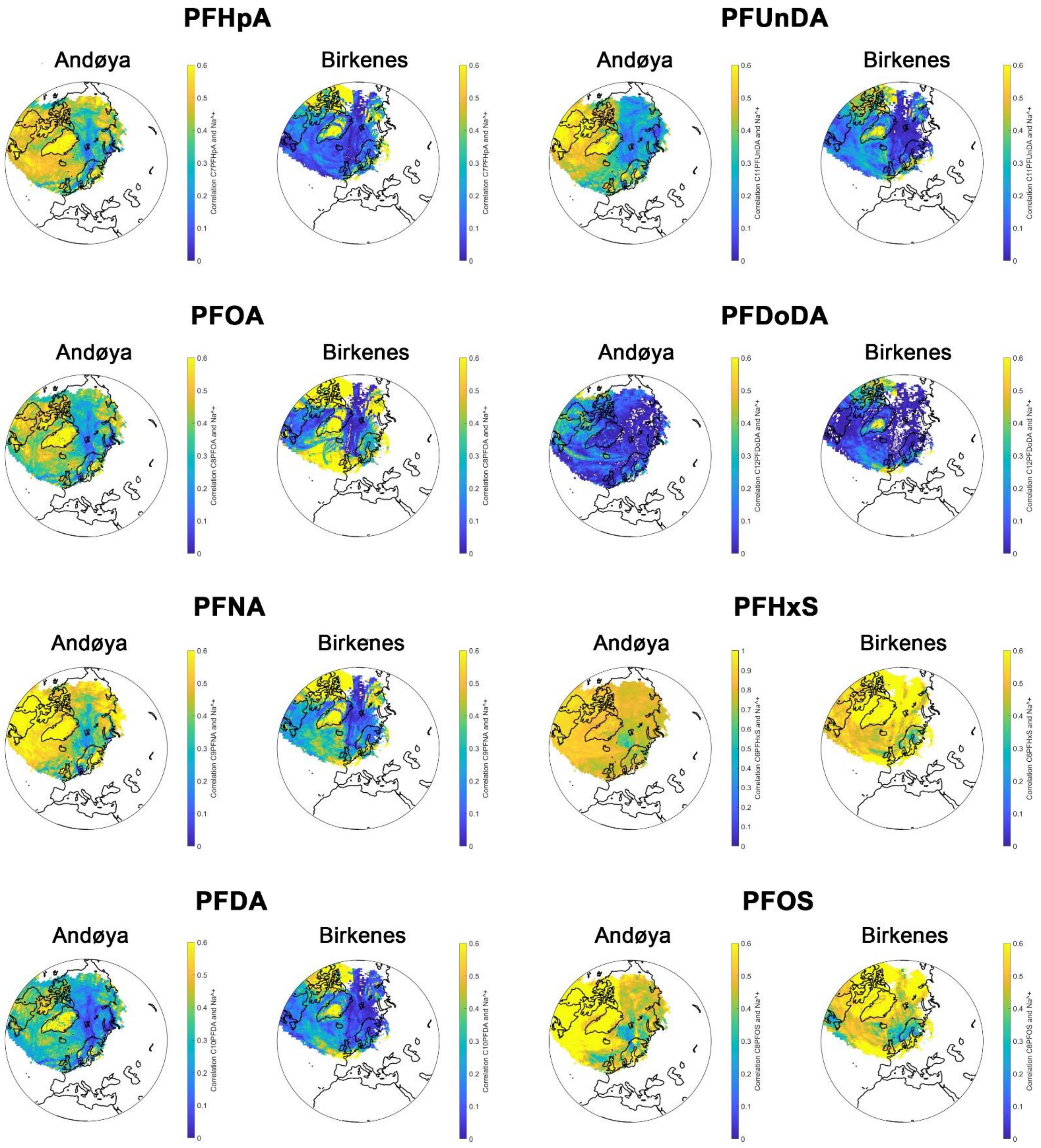


**Figure S6 Correlations between PFAAs and $Na^+$ per grid cell.**

**Table S1. Target compounds.**

| Compound | Acronym | CAS No. | Formula |
|---|---|---|---|
| Perfluoropentanoic acid | PFPeA | 2706-90-3 | C4F9COOH |
| Perfluorohexanoic acid | PFHxA | 307-24-4 | C5F11COOH |
| Perfluoroheptanoic acid | PFHpA | 375-85-9 | C6F13COOH |
| Perfluorooctanoic acid | PFOA | 335-67-1 | C7F15COOH |
| Perfluorononanoic acid | PFNA | 375-95-1 | C8F17COOH |
| Perfluorodecanoic acid | PFDA | 335-76-2 | C9F19COOH |
| Perfluoroundecanoic acid | PFUnDA | 2058-94-8 | C10F21COOH |
| Perfluorododecanoic acid | PFDoDA | 307-55-1 | C11F23COOH |
| Perfluorobutane sulfonic acid | PFBS | 375-73-5 | C4F9SO3H |
| Perfluorohexane sulfonic acid | PFHxS | 355-46-4 | C6F13SO3H |
| Perfluorooctane sulfonic acid | PFOS | 1763-23-1 | C8F17SO3H |

**Table S2. Standards and reagents.**

| Name | | Supplier |
|---|---|---|
| PFPeA | Perfluoro-n-pentanoic acid (>98%) | Wellington Laboratories |
| PFHxA | Perfluoro-n-hexanoic acid (>98%) | Wellington Laboratories |
| PFHpA | Perfluoro-n-heptanoic acid (>98%) | Wellington Laboratories |
| T-PFOA | Technical Ammonium Perfluorooctanoate (>97%) | Wellington Laboratories |
| PFNA | Perfluoro-n-nonanoic acid (>98%) | Wellington Laboratories |
| PFDA | Perfluoro-n-decanoic acid (>98%) | Wellington Laboratories |
| PFUnDA | Perfluoro-n-undecanoic acid (>98%) | Wellington Laboratories |
| PFDoDA | Perfluoro-n-dodecanoic acid (>98%) | Wellington Laboratories |
| L-PFBS | Potassium perfluoro-1-butanesulfonate (>98%) | Wellington Laboratories |
| L-PFHxS | Sodium perfluoro-1-hexanesulfonate (>98%) | Wellington Laboratories |
| T-PFOS | Potassium perfluorooctanesulfonate (Technical Grade, ~80%) | Wellington Laboratories |
| | | |
| $^{13}C_5$-PFPeA | Perfluoro-n-[1,2,3,4,5-$^{13}C_5$]pentanoic acid | Wellington Laboratories |
| $^{13}C_2$-PFHxA | Perfluoro-n-[1,2-$^{13}C_2$]hexanoic acid | Wellington Laboratories |
| $^{13}C_4$-PFHpA | Perfluoro-n-[1,2,3,4-$^{13}C_4$]heptanoic acid | Wellington Laboratories |
| $^{13}C_4$-PFOA | Perfluoro-n-[1,2,3,4-$^{13}C_4$]octanoic acid | Wellington Laboratories |
| $^{13}C_5$-PFNA | Perfluoro-n-[1,2,3,4,5-$^{13}C_5$]nonanoic acid | Wellington Laboratories |
| $^{13}C_2$-PFDA | Perfluoro-n-[1,2-$^{13}C_2$]decanoic acid | Wellington Laboratories |
| $^{13}C_2$-PFUnDA | Perfluoro-n-[1,2-$^{13}C_2$]undecanoic acid | Wellington Laboratories |
| $^{13}C_2$-PFDoDA | Perfluoro-n-[1,2-$^{13}C_2$]dodecanoic acid | Wellington Laboratories |
| $^{13}C_4$-PFBA | Perfluoro-n-[1,2,3,4-$^{13}C_4$]butanoic acid | Wellington Laboratories |
| $^{18}O_2$-PFHxS | Sodium perfluoro-1-hexane[$^{18}O_2$]sulfonate | Wellington Laboratories |
| $^{13}C_4$-PFOS | Sodium perfluoro-1-[1,2,3,4-$^{13}C_4$]octanesulfonate | Wellington Laboratories |
| $^{13}C_8$-PFOA | Perfluoro-n-[$^{13}C_8$]octanoic acid | Wellington Laboratories |
| $^{13}C_8$-PFOS | Sodium perfluoro-[$^{13}C_8$]octanesulfonate | Wellington Laboratories |
| | | |
| Methanol | | Merck |
| Formic acid (98% ACS, Reag. PhEur) | | Merck |
| Ammonium acetate (ProAnalysis) | | Merck |
| Ammonium formate (> 99 %, for HPLC) | | Fluka |
| 25% ammonium hydroxide solution | | Sigma-Aldrich |

**Table S3. The start date, end date, sampling duration and total volume of each sample collected at Andøya and Birkenes.**

| No | Start Date | End Date | Volume ($m^3$) | Duration (hour) | No | Start Date | End Date | Volume ($m^3$) | Duration (hour) |
|---|---|---|---|---|---|---|---|---|---|
| **Andøya** | | | | | | | | | |
| A01 | 2018-04-18 07:22 | 2018-04-20 07:31 | 1490.8 | 48.2 | A30 | 2019-08-14 07:35 | 2019-08-16 07:32 | 1479.1 | 48.0 |
| A02 | 2018-04-25 07:36 | 2018-04-27 07:10 | 1472.7 | 47.6 | A31 | 2019-09-04 07:39 | 2019-09-05 07:36 | 738.8 | 24.0 |
| A03 | 2018-07-11 07:41 | 2018-07-13 07:25 | 1477.9 | 47.7 | A32 | 2019-09-11 07:55 | 2019-09-13 07:53 | 1479.6 | 48.0 |
| A04 | 2018-07-18 07:23 | 2018-07-20 07:24 | 1486.7 | 48.0 | A33 | 2019-09-25 07:48 | 2019-09-27 07:40 | 1476.5 | 47.9 |
| A05 | 2018-08-01 07:34 | 2018-08-03 07:31 | 1484.7 | 48.0 | A34 | 2019-10-02 08:00 | 2019-10-04 08:00 | 1480.6 | 48.0 |
| A06 | 2018-08-08 07:49 | 2018-08-10 07:26 | 1474.3 | 47.6 | A35 | 2019-10-11 13:58 | 2019-10-14 09:58 | 2098.1 | 68.0 |
| A07 | 2018-08-15 07:21 | 2018-08-17 07:14 | 1451.6 | 47.9 | A36 | 2019-10-23 07:28 | 2019-10-25 08:41 | 1518.6 | 49.2 |
| A08 | 2018-08-29 07:35 | 2018-08-31 07:49 | 1493.4 | 48.2 | A37 | 2019-10-30 08:53 | 2019-11-01 08:49 | 1479.0 | 47.9 |
| A09 | 2018-09-05 07:39 | 2018-09-07 07:13 | 1472.8 | 47.6 | A38 | 2019-11-06 08:38 | 2019-11-08 08:43 | 1483.6 | 48.1 |
| A10 | 2018-09-12 07:48 | 2018-09-14 08:13 | 1499.5 | 48.4 | A39 | 2019-11-20 08:47 | 2019-11-22 08:50 | 1482.6 | 48.0 |
| A11 | 2018-09-26 07:31 | 2018-09-28 07:43 | 1492.3 | 48.2 | A40 | 2019-11-27 08:44 | 2019-11-29 08:29 | 1473.3 | 47.8 |
| A12 | 2018-10-03 07:59 | 2018-10-05 07:46 | 1479.5 | 47.8 | A41 | 2019-12-04 08:56 | 2019-12-06 08:36 | 1470.7 | 47.7 |
| A13 | 2018-10-10 07:30 | 2018-10-12 07:30 | 1486.1 | 48.0 | A42 | 2019-12-13 08:47 | 2019-12-16 08:41 | 2218.4 | 71.9 |
| A14 | 2018-10-24 07:35 | 2018-10-26 07:43 | 1490.3 | 48.1 | A43 | 2020-01-10 13:07 | 2020-01-13 08:34 | 2081.1 | 67.5 |
| A15 | 2018-10-31 08:53 | 2018-11-02 09:06 | 1492.9 | 48.2 | A44 | 2020-01-17 08:36 | 2020-01-20 08:34 | 2220.5 | 72.0 |
| A16 | 2018-11-28 08:56 | 2018-11-30 11:31 | 1560.3 | 50.6 | A45 | 2020-01-22 09:15 | 2020-01-24 08:41 | 1463.5 | 47.4 |
| A17 | 2018-12-05 08:30 | 2018-12-07 08:35 | 1483.2 | 48.1 | A46 | 2020-01-29 10:29 | 2020-01-31 09:05 | 1437.8 | 46.6 |
| A18 | 2019-01-30 09:55 | 2019-02-01 09:37 | 1471.4 | 47.7 | A47 | 2020-04-17 12:08 | 2020-04-20 07:48 | 2087.8 | 67.7 |
| A19 | 2019-02-13 08:49 | 2019-02-15 08:34 | 1472.9 | 47.8 | A48 | 2020-04-24 08:01 | 2020-04-27 08:59 | 2251.3 | 73.0 |
| A20 | 2019-02-27 08:48 | 2019-03-01 08:30 | 1471.4 | 47.7 | A49 | 2020-05-08 08:13 | 2020-05-11 07:38 | 2203.5 | 71.4 |
| A21 | 2019-03-20 08:39 | 2019-03-22 10:08 | 1526.4 | 49.5 | A50 | 2020-05-13 07:47 | 2020-05-15 07:48 | 1481.5 | 48.0 |
| A22 | 2019-03-27 15:02 | 2019-03-29 14:28 | 1463.1 | 47.4 | A51 | 2020-06-03 07:42 | 2020-06-05 07:50 | 1485.1 | 48.1 |
| A23 | 2019-05-08 07:27 | 2019-05-10 07:25 | 1479.6 | 48.0 | A52 | 2020-06-10 08:19 | 2020-06-12 09:05 | 1504.7 | 48.8 |
| A24 | 2019-05-22 07:03 | 2019-05-24 07:23 | 1490.9 | 48.3 | A53 | 2020-06-17 07:47 | 2020-06-19 08:05 | 1490.3 | 48.3 |
| A25 | 2019-06-19 08:25 | 2019-06-21 07:48 | 1461.6 | 47.4 | A54 | 2020-06-26 08:19 | 2020-06-29 07:35 | 2198.9 | 71.3 |
| A26 | 2019-07-03 07:26 | 2019-07-05 07:15 | 1475.0 | 47.8 | A55 | 2020-07-01 07:49 | 2020-07-03 07:49 | 1481.0 | 48.0 |
| A27 | 2019-07-17 07:05 | 2019-07-19 06:43 | 1469.3 | 47.6 | A56 | 2020-07-08 08:56 | 2020-07-10 09:07 | 1486.7 | 48.2 |
| A28 | 2019-07-31 07:42 | 2019-08-02 07:41 | 1480.1 | 48.0 | A57 | 2020-07-29 07:37 | 2020-07-31 07:56 | 1490.8 | 48.3 |
| A29 | 2019-08-07 07:48 | 2019-08-09 07:32 | 1472.4 | 47.7 | | | | | |

**Birkenes**

| | | | | | | | | | |
|---|---|---|---|---|---|---|---|---|---|
| B01 | 2018-04-12 04:32 | 2018-04-14 04:29 | 1439.6 | 48.0 | B30 | 2018-09-24 04:29 | 2018-09-26 04:25 | 1438.5 | 47.9 |
| B02 | 2018-04-14 04:33 | 2018-04-16 04:32 | 1423.3 | 48.0 | B31 | 2018-09-29 04:35 | 2018-10-01 04:27 | 1340.6 | 47.9 |
| B03 | 2018-04-21 04:31 | 2018-04-23 04:30 | 1429.2 | 48.0 | B32 | 2018-10-01 04:28 | 2018-10-03 04:20 | 1421.2 | 47.9 |
| B04 | 2018-04-23 04:33 | 2018-04-25 04:25 | 1422.5 | 47.9 | B33 | 2018-10-06 04:35 | 2018-10-08 04:21 | 1343.5 | 47.8 |
| B05 | 2018-04-26 04:29 | 2018-04-28 04:32 | 1428.8 | 48.0 | B34 | 2018-10-08 04:22 | 2018-10-10 04:21 | 1421.6 | 48.0 |
| B06 | 2018-04-28 04:39 | 2018-04-30 04:30 | 1424.7 | 47.9 | B35 | 2018-10-13 04:37 | 2018-10-15 04:30 | 1326.5 | 47.9 |
| B07 | 2018-04-29 04:36 | 2018-05-01 04:33 | 1428.2 | 48.0 | B36 | 2019-09-09 04:28 | 2019-09-11 04:31 | 1421.8 | 48.0 |
| B08 | 2018-05-05 04:34 | 2018-05-07 04:25 | 1421.1 | 47.9 | B37 | 2019-09-11 04:33 | 2019-09-13 04:32 | 1417.3 | 48.0 |
| B09 | 2018-05-07 04:29 | 2018-05-09 04:26 | 1414.1 | 48.0 | B38 | 2019-09-16 04:25 | 2019-09-18 04:28 | 1423.2 | 48.0 |
| B10 | 2018-05-12 04:34 | 2018-05-14 04:25 | 1415.9 | 47.9 | B39 | 2019-09-23 04:32 | 2019-09-25 04:28 | 1425.1 | 47.9 |
| B11 | 2018-05-14 04:29 | 2018-05-16 04:19 | 1402.8 | 47.8 | B40 | 2019-09-25 04:30 | 2019-09-27 04:32 | 1422.8 | 48.0 |
| B12 | 2018-05-19 04:32 | 2018-05-21 04:29 | 1417.8 | 48.0 | B41 | 2019-09-30 04:23 | 2019-10-02 04:37 | 1420.9 | 48.2 |
| B13 | 2018-05-21 04:34 | 2018-05-23 04:23 | 1412.1 | 47.8 | B42 | 2019-10-07 04:21 | 2019-10-09 04:30 | 1435.1 | 48.1 |
| B14 | 2018-05-26 04:33 | 2018-05-28 04:25 | 1403.6 | 47.9 | B43 | 2019-10-09 04:32 | 2019-10-11 04:32 | 1415.2 | 48.0 |
| B15 | 2018-05-28 04:30 | 2018-05-30 04:19 | 1408.7 | 47.8 | B44 | 2019-10-14 04:26 | 2019-10-16 04:25 | 1428.1 | 48.0 |
| B16 | 2018-06-02 04:32 | 2018-06-04 04:27 | 1385.4 | 47.9 | B45 | 2019-10-16 04:28 | 2019-10-18 04:26 | 1341.1 | 48.0 |
| B17 | 2018-06-04 04:30 | 2018-06-06 04:28 | 1409.3 | 48.0 | B46 | 2019-10-21 04:36 | 2019-10-23 04:27 | 1429.1 | 47.9 |
| B18 | 2018-07-25 04:37 | 2018-07-27 04:29 | 1313.6 | 47.9 | B47 | 2019-10-23 04:29 | 2019-10-25 04:31 | 1421.1 | 48.0 |
| B19 | 2018-07-28 04:31 | 2018-07-30 04:24 | 1311.7 | 47.9 | B48 | 2019-10-28 05:30 | 2019-10-30 05:31 | 1453.4 | 48.0 |
| B20 | 2018-08-04 04:32 | 2018-08-06 04:27 | 1319.1 | 47.9 | B49 | 2019-10-30 05:40 | 2019-11-01 05:30 | 1353.4 | 47.8 |
| B21 | 2018-08-11 04:26 | 2018-08-13 04:21 | 1326.8 | 47.9 | B50 | 2019-11-02 05:41 | 2019-11-04 05:36 | 1341.9 | 47.9 |
| B22 | 2018-08-18 04:32 | 2018-08-20 04:21 | 1326.9 | 47.8 | B51 | 2019-11-09 05:41 | 2019-11-11 05:31 | 1357.4 | 47.8 |
| B23 | 2018-08-25 04:30 | 2018-08-27 04:20 | 1325.9 | 47.8 | B52 | 2019-11-13 05:35 | 2019-11-15 05:33 | 1349.7 | 48.0 |
| B24 | 2018-09-01 04:29 | 2018-09-03 04:21 | 1340.0 | 47.9 | B53 | 2019-11-16 05:38 | 2019-11-18 05:39 | 1353.2 | 48.0 |
| B25 | 2018-09-08 04:30 | 2018-09-10 04:22 | 1330.5 | 47.9 | B54 | 2019-11-23 05:34 | 2019-11-25 05:26 | 1350.7 | 47.9 |
| B26 | 2018-09-10 04:26 | 2018-09-12 04:22 | 1417.5 | 47.9 | B55 | 2019-11-27 05:32 | 2019-11-29 05:32 | 1343.7 | 48.0 |
| B27 | 2018-09-15 04:37 | 2018-09-17 04:25 | 1335.7 | 47.8 | B56 | 2019-11-30 05:41 | 2019-12-02 05:26 | 1360.6 | 47.8 |
| B28 | 2018-09-17 04:27 | 2018-09-19 04:19 | 1406.3 | 47.9 | B57 | 2019-12-07 05:41 | 2019-12-09 05:30 | 1330.9 | 47.8 |
| B29 | 2018-09-22 04:50 | 2018-09-24 04:27 | 1329.5 | 47.6 | B58 | 2019-12-11 05:40 | 2019-12-13 05:36 | 1339.9 | 47.9 |

**Table S4. Laboratory blanks, field blanks, MDLs, IS recovery and result of spike recovery test.**

| Analytes | Lab blanks (mean±SD, pg/QFF) | MDL (mean+3×SD, pg/QFF) | Field blanks[a] (mean±SD (DF [b]), pg/QFF) | | Spike recovery (%) | | IS recovery (%) |
|---|---|---|---|---|---|---|---|
| | | | Andøya (n=9) | Birkenes (n=7) | 30 pg | 300 pg | |
| C6-PFHxA | 13±2 | 19 | 20 (11%) | 27±11 (43%) | 102±26 | 87±11 | 33±19 |
| C7-PFHpA | 3±1 | 6 | 7 (11%) | <MDL (0%) | 102±8 | 96±10 | 43±19 |
| C8-PFOA | 6±1 | 9 | 12±1 (56%) | 12±2 (57%) | 84±18 | 88±11 | 62±17 |
| C9-PFNA | 3±1 | 6 | 8 (11%) | <MDL (0%) | 102±8 | 92±11 | 56±17 |
| C10-PFDA | 4±2 | 10 | 11 (11%) | <MDL (0%) | 99±12 | 93±12 | 82±32 |
| C11-PFUnDA | 3±1 | 6 | <MDL (0%) | <MDL (0%) | 102±12 | 90±13 | 67±50 |
| C12-PFDoDA | 3±2 | 9 | 10±1 (33%) | <MDL (0%) | 101±5 | 86±11 | 42±45 |
| C4-PFBS | 4±2 | 10 | 14 (11%) | 15 (11%) | 88±20 | 79±12 | n.a. [c] |
| C6-PFHxS | 3±1 | 6 | <MDL (0%) | <MDL (0%) | 106±8 | 90±12 | 70±15 |
| C8-PFOS | 3±1 | 6 | <MDL (0%) | <MDL (0%) | 91±17 | 85±12 | 59±12 |
| Na | n.d. [c] | 13 μg/QFF [e] | <MDL (0%) | <MDL (0%) | n.a. [d] | n.a. | n.a. |
| Mg | n.d. | 7 μg/QFF [e] | <MDL (0%) | <MDL (0%) | n.a. | n.a. | n.a. |

[a] Field blanks shown here were not corrected by the mean value of laboratory blanks.
[b] DF – detection frequency
[c] n.a. – not applicable.
[d] MDLs of Na and Mg were determined by 3 times signal-to-noise level

**Table S5. Results of Pearson correlation and orthogonal linear regressions ($\log_{10}[PFAA]=k\cdot\log_{10}[Na]+b$) between PFAA concentrations and $Na^+$ concentration in the samples. The significance level of the Pearson correlation coefficient (r) is indicated by the number of asterisks (*$p$<0.05, **$p$<0.01, ***$p$<0.001).**

| | All samples >MDL | | | | All samples >MDL and values <MDL are substituted by ½ MDL | | | | Samples with PFAA/Na between the 5th and 95th percentile | | | |
|---|---|---|---|---|---|---|---|---|---|---|---|---|
| Compound | n | k | b | r | n | K | b | r | n | k | b | r |
| **Andoya** | | | | | | | | | | | | |
| C6-PFHxA | 28 | 2.731 | -0.997 | 0.338 | 57 | 1.992 | -1.209 | 0.590*** | 24 | 1.675 | -1.185 | 0.498* |
| C7-PFHpA | 42 | 0.845 | -1.114 | 0.539*** | 57 | 2.045 | -0.863 | 0.678*** | 36 | 0.966 | -1.097 | 0.706*** |
| C8-PFOA | 52 | 1.183 | -0.479 | 0.604*** | 57 | 2.045 | -0.216 | 0.603*** | 46 | 1.056 | -0.556 | 0.774*** |
| C9-PFNA | 56 | 0.909 | -0.571 | 0.549*** | 57 | 1.193 | -0.474 | 0.531*** | 50 | 0.972 | -0.562 | 0.75*** |
| C10-PFDA | 57 | 1.046 | -0.620 | 0.516*** | 57 | 1.046 | -0.620 | 0.516*** | 51 | 0.887 | -0.701 | 0.696*** |
| C11-PFUnDA | 53 | 0.629 | -1.206 | 0.363** | 57 | 2.476 | -0.513 | 0.129 | 47 | 0.682 | -1.166 | 0.63*** |
| C12-PFDoDA | 33 | 0.611 | -1.009 | 0.426* | 57 | -8.175 | -5.476 | -0.138 | 29 | 0.554 | -1.041 | 0.597*** |
| C6-PFHxS | 44 | 0.590 | -2.088 | 0.73*** | 57 | 0.769 | -2.081 | 0.794*** | 38 | 0.788 | -2.041 | 0.786*** |
| C8-PFOS | 56 | 0.713 | -1.216 | 0.559*** | 57 | 0.819 | -1.181 | 0.603*** | 50 | 0.868 | -1.163 | 0.698*** |
| **Birkenes** | | | | | | | | | | | | |
| C6-PFHxA | 6 | -0.543 | -1.676 | -0.722 | 58 | 0.021 | -2.502 | 0.017 | 4 | -1.049 | -1.750 | -0.656 |
| C7-PFHpA | 37 | 0.164 | -1.431 | 0.087 | 58 | 1.446 | -0.876 | 0.343** | 34 | 0.441 | -1.283 | 0.319 |
| C8-PFOA | 52 | 0.285 | -0.976 | 0.196 | 58 | 0.743 | -0.716 | 0.449*** | 48 | 0.419 | -0.887 | 0.464*** |
| C9-PFNA | 52 | 0.295 | -0.853 | 0.272 | 58 | 0.819 | -0.566 | 0.366** | 48 | 0.453 | -0.748 | 0.463*** |
| C10-PFDA | 54 | 0.142 | -1.416 | 0.066 | 58 | 0.412 | -1.254 | 0.313* | 50 | 0.449 | -1.197 | 0.218 |
| C11-PFUnDA | 54 | 0.013 | -1.604 | 0.003 | 58 | 0.488 | -1.304 | 0.263* | 50 | 0.596 | -1.195 | 0.168 |
| C12-PFDoDA | 31 | -0.212 | -1.914 | -0.163 | 58 | 0.352 | -1.950 | 0.078 | 27 | 0.185 | -1.661 | 0.158 |
| C6-PFHxS | 46 | 0.612 | -1.756 | 0.633*** | 58 | 0.661 | -1.780 | 0.718*** | 40 | 0.745 | -1.683 | 0.708*** |
| C8-PFOS | 56 | 0.461 | -1.064 | 0.631*** | 58 | 0.412 | -1.091 | 0.654*** | 52 | 0.527 | -1.004 | 0.653*** |

**Table S6. Detection frequencies and concentration ranges of the analytes.**

| Analyte | Andøya (*n*=57) DF (%) | Median ($pg\ m^{-3}$) | Range ($pg\ m^{-3}$) | Mean±SD[a] ($pg\ m^{-3}$) | Birkenes (*n*=58) DF (%) | Median ($pg\ m^{-3}$) | Range ($pg\ m^{-3}$) | Mean±SD[a] ($pg\ m^{-3}$) |
|---|---|---|---|---|---|---|---|---|
| C6-PFHxA | 49 | <0.004 | <0.004 - 0.285 | 0.038±0.058 | 10 | <0.004 | <0.004 - 0.191 | 0.009±0.03 |
| C7-PFHpA | 74 | 0.033 | <0.004 - 0.228 | 0.046±0.055 | 67 | 0.020 | <0.004 - 0.257 | 0.037±0.049 |
| C8-PFOA | 91 | 0.108 | <0.003 - 1.28 | 0.19±0.24 | 93 | 0.068 | <0.003 - 0.811 | 0.091±0.11 |
| C9-PFNA | 98 | 0.138 | <0.004 - 0.467 | 0.16±0.12 | 93 | 0.082 | <0.004 - 0.555 | 0.12±0.11 |
| C10-PFDA | 100 | 0.104 | 0.007 - 1.01 | 0.14±0.19 | 97 | 0.026 | <0.007 - 0.133 | 0.044±0.039 |
| C11-PFUnDA | 93 | 0.041 | <0.004 - 0.112 | 0.043±0.028 | 97 | 0.021 | <0.004 - 0.242 | 0.039±0.043 |
| C12-PFDoDA | 58 | 0.046 | <0.004 - 0.331 | 0.068±0.076 | 53 | 0.007 | <0.004 - 0.112 | 0.016±0.02 |
| C4-PFBS | 14 | <0.007 | <0.007 - 0.035 | 0.004±0.006 | 9 | <0.007 | <0.007 - 0.028 | 0.004±0.004 |
| C6-PFHxS | 77 | 0.004 | <0.004 - 0.019 | 0.005±0.004 | 79 | 0.006 | <0.004 - 0.07 | 0.009±0.013 |
| C8-PFOS | 98 | 0.03 | <0.004 - 0.144 | 0.040±0.029 | 100 | 0.039 | 0.006 - 0.392 | 0.055±0.061 |
| Na ($\mu g\ m^{-3}$) | 100 | 0.428 | 0.022 - 3.18 | 0.58±0.63 | 97 | 0.21 | <0.009 - 1.73 | 0.361±0.409 |
| Mg ($\mu g\ m^{-3}$) | 98 | 0.068 | <0.005 - 0.451 | 0.088±0.086 | 90 | 0.04 | <0.005 - 0.267 | 0.06±0.06 |

[a] values <MDL were replaced by ½MDL when calculating mean and SD.

**Table S7. Results of Pearson correlation between PFAA concentrations and $Na^{+}$ concentration in the summer and winter samples. The significance level of the Pearson correlation coefficient (r) is indicated by the number of asterisks (**p*<0.05, ***p*<0.01, ****p*<0.001). The data is fitted by $log_{10}$[PFAA]=$log_{10}$(*k*×[$Na^{+}$]+*b*).**

| | All samples | | | | Winter | | | | Summer | | | |
|---|---|---|---|---|---|---|---|---|---|---|---|---|
| Compound | n | k | b | r | n | K | b | r | n | k | b | r |
| **Andoya** | | | | | | | | | | | | |
| C6-PFHxA | 24 | 0.033 | 0.0089 | 0.498* | 12 | 0.079 | 0.0000 | 0.668* | 12 | 0.014 | 0.0107 | 0.712** |
| C7-PFHpA | 36 | 0.056 | 0.012 | 0.706*** | 16 | 0.089 | 0.0033 | 0.744*** | 20 | 0.039 | 0.0162 | 0.757*** |
| C8-PFOA | 46 | 0.23 | 0.011 | 0.774*** | 17 | 0.345 | 0.0000 | 0.706** | 29 | 0.170 | 0.0149 | 0.843*** |
| C9-PFNA | 50 | 0.23 | 0.016 | 0.750*** | 19 | 0.265 | 0.0123 | 0.718*** | 31 | 0.198 | 0.0186 | 0.772*** |
| C10-PFDA | 51 | 0.14 | 0.023 | 0.696*** | 18 | 0.226 | 0.0028 | 0.743*** | 33 | 0.102 | 0.0306 | 0.716*** |
| C11-PFUnDA | 47 | 0.041 | 0.016 | 0.630*** | 18 | 0.064 | 0.0104 | 0.57* | 29 | 0.030 | 0.0188 | 0.707*** |
| C12-PFDoDA | 29 | 0.051 | 0.027 | 0.597*** | 7 | 0.123 | 0.0111 | 0.661 | 22 | 0.037 | 0.0288 | 0.614** |
| C6-PFHxS | 38 | 0.0061 | 0.0021 | 0.786*** | 21 | 0.006 | 0.0029 | 0.631** | 17 | 0.006 | 0.0011 | 0.936*** |
| C8-PFOS | 50 | 0.050 | 0.0090 | 0.698*** | 23 | 0.056 | 0.0118 | 0.617** | 27 | 0.040 | 0.0086 | 0.736*** |
| **Birkenes** | | | | | | | | | | | | |
| C6-PFHxA | 4 | n.a. | n.a. | n.a. | 3 | n.a. | n.a. | n.a. | 1 | n.a. | n.a. | n.a. |
| C7-PFHpA | 34 | 0.0185 | 0.0227 | 0.319 | 15 | 0.014 | 0.0150 | 0.303 | 19 | 0.043 | 0.0250 | 0.526* |
| C8-PFOA | 48 | 0.0782 | 0.0448 | 0.464*** | 20 | 0.085 | 0.0271 | 0.681*** | 28 | 0.091 | 0.0535 | 0.463* |
| C9-PFNA | 48 | 0.1029 | 0.0566 | 0.463*** | 20 | 0.103 | 0.0234 | 0.711*** | 28 | 0.105 | 0.0921 | 0.453* |
| C10-PFDA | 50 | 0.0220 | 0.0247 | 0.218 | 20 | 0.024 | 0.0098 | 0.463* | 30 | 0.023 | 0.0404 | 0.247 |
| C11-PFUnDA | 50 | 0.0160 | 0.0203 | 0.168 | 20 | 0.014 | 0.0072 | 0.41 | 30 | 0.020 | 0.0359 | 0.239 |
| C12-PFDoDA | 27 | 0.0062 | 0.0142 | 0.158 | 6 | 0.008 | 0.0095 | 0.206 | 21 | 0.006 | 0.0155 | 0.191 |
| C6-PFHxS | 40 | 0.0144 | 0.0033 | 0.708*** | 16 | 0.010 | 0.0042 | 0.644** | 24 | 0.019 | 0.0025 | 0.757*** |
| C8-PFOS | 52 | 0.0728 | 0.0219 | 0.653*** | 22 | 0.085 | 0.0162 | 0.764*** | 30 | 0.063 | 0.0264 | 0.544** |

**Table S8. Enrichment factors from the previous laboratory study using a SSA chamber.**[3]

| Compound | Enrichment factors in particles with d<10μm ($EF_{10}$) | | Enrichment factors in particleswith d<1.5μm ($EF_{1.5}$) | |
|---|---|---|---|---|
| | Mean | SD | Mean | SD |
| C6-PFHxA | 325 | 98 | 155 | 114 |
| C7-PFHpA | 1952 | 446 | 1379 | 1025 |
| C8-PFOA | 4393 | 763 | 4605 | 1779 |
| C9-PFNA | 6578 | 882 | 10618 | 3677 |
| C10-PFDA | 7878 | 1064 | 17570 | 3638 |
| C11-PFUnDA | 8582 | 911 | 22572 | 2992 |
| C12-PFDoDA | 8620 | 1953 | 24958 | 5760 |
| C6-PFHxS | 4845 | 392 | 13659 | 1044 |
| C8-PFOS | 9571 | 1387 | 28588 | 4619 |

**Table S9**. **Medians (ng L$^{-1}$) and sample numbers (#) for all coastal and open ocean sites from Muir and Miaz.[4] * indicates value at detection limit.**

| Region | period | PFHxA | | PFHpA | | PFOA | | PFNA | | PFDA | | PFUnDA | | PFDoDA | | PFHxS | | PFOS | |
|---|---|---|---|---|---|---|---|---|---|---|---|---|---|---|---|---|---|---|---|
| | | median | # | median | # | median | # | median | # | median | # | median | # | median | # | median | # | median | # |
| Arctic Sea | 2000 - 2009 | 0.01 | 96 | 0.0175 | 68 | 0.0498 | 122 | 0.0097 | 96 | 0.0023 | 33 | 0.0036 | 33 | 0.0014* | 33 | 0.008 | 120 | 0.0105 | 122 |
| | 2010 - 2014 | 0.0014* | 63 | 0.014 | 63 | 0.043 | 65 | 0.0186 | 63 | 0.0014* | 77 | 0.0014* | 63 | 0.0014* | 57 | 0.0035* | 63 | 0.021 | 82 |
| | 2015 - 2019 | 0.051 | 31 | 0.028 | 31 | 0.054 | 31 | 0.032 | 31 | 0.007 | 31 | 0.0061 | 31 | 0.0014* | 31 | 0.0035* | 31 | 0.025 | 31 |
| North Atlantic | 2000 - 2009 | 0.024 | 117 | 0.018 | 117 | 0.08 | 141 | 0.025 | 117 | 0.0054 | 67 | 0.0023 | 51 | 0.0014* | 51 | 0.0076 | 75 | 0.043 | 173 |
| | 2010 - 2014 | 0.079 | 85 | 0.0655 | 68 | 0.103 | 85 | 0.044 | 85 | 0.0335 | 68 | --- | --- | --- | --- | 0.043 | 85 | 0.0749 | 85 |
| | 2015 - 2019 | --- | --- | --- | --- | --- | --- | --- | --- | --- | --- | --- | --- | --- | --- | --- | --- | --- | --- |
| North Sea | 2000 - 2009 | 0.55 | 58 | 0.35 | 57 | 0.72 | 111 | 0.11 | 58 | 0.02 | 57 | 0.0014 | 15 | 0.0014* | 15 | 0.0915 | 104 | 0.7 | 199 |
| | 2010 - 2014 | 0.47 | 107 | 0.81 | 25 | 1.09 | 107 | 0.05 | 107 | 0.02 | 107 | 0.0014* | 107 | 0.0003* | 25 | 0.25 | 107 | 0.31 | 107 |
| | 2015 - 2019 | 0.77 | 43 | 0.23 | 43 | 0.45 | 43 | 0.0014* | 43 | 0.017 | 4 | 0.0014* | 43 | 0.0055 | 4 | 0.14 | 43 | 0.054 | 51 |
| Baltic Sea | 2000 - 2009 | 14 | 88 | 0.12 | 82 | 0.42 | 97 | 0.2725 | 88 | 0.0014* | 82 | 0.0014* | 8 | 0.0014* | 82 | 0.09 | 97 | 0.21 | 107 |
| | 2010 - 2014 | --- | --- | 0.0014* | 18 | 0.52 | 18 | 1.15 | 18 | 0.0014* | 18 | --- | --- | 0.0014* | 18 | 0.42 | 18 | 0.73 | 18 |
| | 2015 - 2019 | 0.25 | 31 | 0.17 | 31 | 0.28 | 55 | 0.086 | 31 | --- | --- | 0.0014* | 31 | --- | --- | 0.2 | 31 | 0.2 | 75 |

**Table S10. Estimated annual global PFOA and PFOS emissions via SSA.**

| | | Low | Median | High |
|---|---|---|---|---|
| PFOA | $C_{SSA}$ = 0.23 pg μg$^{-1}$ $Na^{+}$) | | | |
| PFOS | $C_{SSA}$ = 0.050 pg μg$^{-1}$ $Na^{+}$) | | | |
| Global emission based on Textor et al.[5] | | | | |
| | SSA ($10^{12}$ kg yr$^{-1}$) | 3.65 | 6.25 | 9.7 |
| | PFOA (tonnes yr$^{-1}$) | 258 | 442 | 686 |
| | PFOS (tonnes yr$^{-1}$) | 56 | 96 | 149 |
| Global emission based on Gliß et al.[6] | | | | |
| | SSA ($10^{12}$ kg yr$^{-1}$) | 3.65 | 4.98 | 6.62 |
| | PFOA (tonnes yr$^{-1}$) | 258 | 352 | 468 |
| | PFOS (tonnes yr$^{-1}$) | 56 | 77 | 102 |
| Modelling result by Johansson et al.[7] | | | | |
| PFOA | Global emission (tonnes yr$^{-1}$) | 23 | 122 | 506 |
| PFOS | Global emission (tonnes yr$^{-1}$) | 42 | 183 | 810 |

## References


(1) Benskin, J. P.; Ikonomou, M. G.; Woudneh, M. B.; Cosgrove, J. R. Rapid Characterization of Perfluoralkyl Carboxylate, Sulfonate, and Sulfonamide Isomers by High-Performance Liquid Chromatography–Tandem Mass Spectrometry. *J. Chromatogr. A* **2012**, *1247*, 165–170. https://doi.org/10.1016/j.chroma.2012.05.077.

(2) Lewis, E. R.; Schwartz, S. E. *Sea Salt Aerosol Production: Mechanisms, Methods, Measurements and Models*; Geophysical Monograph Series; AGU: Washington D.C., 2004; Vol. 152.

(3) Sha, B.; Johansson, J. H.; Benskin, J. P.; Cousins, I. T.; Salter, M. E. Influence of Water Concentrations of Perfluoroalkyl Acids (PFAAs) on Their Size-Resolved Enrichment in Nascent Sea Spray Aerosols. *Environ. Sci. Technol.* **2020**. https://doi.org/10.1021/acs.est.0c03804.

(4) Muir, D.; Miaz, L. T. Spatial and Temporal Trends of Perfluoroalkyl Substances in Global Ocean and Coastal Waters. *Environ. Sci. Technol.* **2021**. https://doi.org/10.1021/acs.est.0c08035.

(5) Textor, C.; Schulz, M.; Guibert, S.; Kinne, S.; Balkanski, Y.; Bauer, S.; Berntsen, T.; Berglen, T.; Boucher, O.; Chin, M.; Dentener, F.; Diehl, T.; Easter, R.; Feichter, H.; Fillmore, D.; Ghan, S.; Ginoux, P.; Gong, S.; Grini, A.; Hendricks, J.; Horowitz, L.; Huang, P.; Isaksen, I.; Iversen, I.; Kloster, S.; Koch, D.; Kirkevåg, A.; Kristjansson, J. E.; Krol, M.; Lauer, A.; Lamarque, J. F.; Liu, X.; Montanaro, V.; Myhre, G.; Penner, J.; Pitari, G.; Reddy, S.; Seland, Ø.; Stier, P.; Takemura, T.; Tie, X. Analysis and Quantification of the Diversities of Aerosol Life Cycles within AeroCom. *Atmospheric Chem. Phys.* **2006**, *6* (7), 1777–1813. https://doi.org/10.5194/acp-6-1777-2006.

(6) Gliß, J.; Mortier, A.; Schulz, M.; Andrews, E.; Balkanski, Y.; Bauer, S. E.; Benedictow, A. M. K.; Bian, H.; Checa-Garcia, R.; Chin, M.; Ginoux, P.; Griesfeller, J. J.; Heckel, A.; Kipling, Z.; Kirkevåg, A.; Kokkola, H.; Laj, P.; Le Sager, P.; Lund, M. T.; Lund Myhre, C.; Matsui, H.; Myhre, G.; Neubauer, D.; van Noije, T.; North, P.; Olivié, D. J. L.; Rémy, S.; Sogacheva, L.; Takemura, T.; Tsigaridis, K.; Tsyro, S. G. AeroCom Phase III Multi-Model Evaluation of the Aerosol Life Cycle and Optical Properties Using Ground- and Space-Based Remote Sensing as Well as Surface in Situ Observations. *Atmospheric Chem. Phys.* **2021**, *21* (1), 87–128. https://doi.org/10.5194/acp-21-87-2021.

(7) Johansson, J. H.; Salter, M. E.; Navarro, J. C. A.; Leck, C.; Nilsson, D. E.; Cousins, I. T. Global Transport of Perfluoroalkyl Acids via Sea Spray Aerosol. *Environ. Sci. Process. Impacts* **2019**, *21* (4), 635–649. https://doi.org/10.1039/C8EM00525G.
